# Genome-wide gene expression changes of *Pseudomonas veronii* 1YdBTEX2 during bioaugmentation in polluted soils

**DOI:** 10.1186/s40793-021-00378-x

**Published:** 2021-04-29

**Authors:** Marian Morales, Vladimir Sentchilo, Noushin Hadadi, Jan Roelof van der Meer

**Affiliations:** Department of Fundamental Microbiology, University of Lausanne, 1015 Lausanne, Switzerland

**Keywords:** RNAseq, Inoculation, Invasion, Survival

## Abstract

**Background:**

Bioaugmentation aims to use the capacities of specific bacterial strains inoculated into sites to enhance pollutant biodegradation. Bioaugmentation results have been mixed, which has been attributed to poor inoculant growth and survival in the field, and, consequently, moderate catalytic performance. However, our understanding of biodegradation activity mostly comes from experiments conducted under laboratory conditions, and the processes occurring during adaptation and invasion of inoculants into complex environmental microbiomes remain poorly known. The main aim of this work was thus to study the specific and different cellular reactions of an inoculant for bioaugmentation during adaptation, growth and survival in natural clean and contaminated non-sterile soils, in order to better understand factors limiting bioaugmentation.

**Results:**

As inoculant we focused on the monoaromatic compound-degrading bacterium *Pseudomonas veronii* 1YdBTEX2. The strain proliferated in all but one soil types in presence and in absence of exogenously added toluene. RNAseq and differential genome-wide gene expression analysis illustrated both a range of common soil responses such as increased nutrient scavenging and recycling, expression of defense mechanisms, as well as environment-specific reactions, notably osmoprotection and metal homeostasis. The core metabolism of *P. veronii* remained remarkably constant during exponential growth irrespective of the environment, with slight changes in cofactor regeneration pathways, possibly needed for balancing defense reactions.

**Conclusions:**

*P. veronii* displayed a versatile global program, enabling it to adapt to a variety of soil environments in the presence and even in absence of its target pollutant toluene. Our results thus challenge the widely perceived dogma of poor survival and growth of exogenous inoculants in complex microbial ecosystems such as soil and provide a further basis to developing successful bioaugmentation strategies.

**Supplementary Information:**

The online version contains supplementary material available at 10.1186/s40793-021-00378-x.

## Background

The recovery and restoration of soils polluted by organic compounds may be enhanced by introducing specific biodegrader bacteria [1–4]. This process, named bioaugmentation, relies on individual or mixtures of inoculant strains with specific metabolic pathways capable to degrade particular organic compounds, to invade, survive and propagate in the contaminated environment at the expense of the degraded pollutant [2, 5–8]. Inoculation attempts frequently do not achieve the intended success and the inoculated strains either do not survive and multiply, or do not display their catabolic properties [9–11]. Whereas most studies have addressed very practical aspects of improving bioaugmentation success, e.g., through strain formulations [3, 12] or process management [1], there is a basic lack of understanding of the factors determining establishment, growth and survival of exogenous strains inside existing microbial ecosystems. Bioaugmentation in that respect is similar to, for example, application of probiotic bacteria in gut systems [13]. And, in a way, the strategies for controlled species growth may relate to those deployed by many pathogenic bacteria to invade native microbiota [14]. Whereas the questions of controlled invasion are old [15], we believe one can learn more about the process and its limitations from the bacteria themselves, how they perceive the transition from sterile culture medium into non-sterile contaminated sites, and which factors they express specifically during growth and maintenance. Such knowledge may help to define specific process conditions favoring controlled growth within existing communities, and increase future success of bioaugmentation.

Previously, we suggested that more attention should be given to the combination of factors representing the actual expected conditions at polluted sites [16], and the molecular and functional strategies displayed by biodegrader bacteria during inoculation under near-field conditions. For example, Moreno et al., [17] found that one-third of the genes of the dibenzofuran-degrading bacterium *Sphingomonas wittichii* RW1 express differently during transition and growth in (polluted) sand as compared to liquid growth with the same carbon substrate [17]. A complementary study done on the same strain using transposon scanning revealed a wide range of genes with selective effects under “soil-specific” conditions [18]. This suggested the existence and importance of many functions specific for survival in soil conditions, although their general nature has remained unclear [18]. In order to understand whether global adaptive responses to soil environments are conserved, we compared transcriptomic changes in *S. wittichii* with those of *Pseudomonas veronii* strain 1YdBTEX2, isolated from sites contaminated with aromatic compounds [19], during the transition from liquid growth media to sandy soil [20]. Similarly to *S. wittichii* RW1, inoculation into sandy soil provoked a major reorganization in global gene expression of *P. veronii* 1YdBTEX2, implicating one-third of all genes, but with very few pathways and biological processes in common between the two [20].

To complement previous studies on *P. veronii* 1YdBTEX2 gene expression changes during adaptation to a soil environment we focus here on its growth and establishment. The questions we aimed to address here were whether *P. veronii* would be capable to grow and survive in natural non-sterile soils, and whether this would be dependent on the presence of contaminated material or specific added carbon substrate (toluene). We further aimed to understand whether *P. veronii* would react differently in response to soil type and whether its cellular reactions could be revealed from global gene expression changes. In our experimental design, we inoculated *P. veronii* and measured population growth in three different natural non-sterile soils, in comparison to regular liquid culture and inert silica matrix, in the presence or absence of externally added toluene or in historically contaminated material with (polycyclic) aromatic hydrocarbons. Community RNA was extracted in exponential phase of *P. veronii* growth and in stationary phase, which was reverse-transcribed, ribosomal RNA depleted, and sequenced (RNA-seq). Adaptation and cellular reactions of *P. veronii* under different conditions were interpreted from a combination of global tools, including gene ontology (GO) [21] and cluster of orthologous groups (COG) assignments of biological processes and pathways [22], a previously constructed genome-scale model of *P. veronii* 1YdBTEX2 (iPsvr) [23], as well as from detailed individual gene or operon annotation information. Transcriptome analysis pointed to a variety of common and specific adaptations in soil environments, but a surprisingly conserved core metabolic expression in exponential phase irrespective of the growth environment.

## Methods

### Media and general culturing conditions of *P. veronii*

*P. veronii* is a toluene, benzene and *m*- and *p*-xylene degrading bacterium originating from contaminated soil [19]. To more easily count *P. veronii* colony-forming units (CFUs) in soil, we used a derivative with a mini-Tn*5* insertion constitutively expressing the green fluorescent protein (GFP) from the P_*circ*_ promoter of the ICE*clc* element [24]. We found no effect on toluene growth in liquid medium between wild-type *P. veronii* and the tagged strain (laboratory strain number 3381, Supplementary Figure 1). For all transcriptomic experiments we used the untagged wild-type strain (lab strain number 3371). *P. veronii* was plated freshly for each experiment from a − 80 °C glycerol stock on nutrient agar (Oxoid), and was grown for 48 h at 30 °C*. P. veronii* colonies were restreaked on 21C-type minimal medium (MM) [25] agar plates, which were incubated in a 10-L closed jar for 72 h at room temperature with 0.5 ml pure toluene as sole carbon and energy source dosed through the vapor phase from an open tube placed in the jar. Liquid suspended precultures were prepared in MM with 10 mM succinate, starting from a single toluene-grown colony, which was incubated for 24 h at 30 °C with rotatory shaking at 180 rpm. In the case of liquid growth experiments, freshly grown precultures were diluted 1:100 (*v/v*) in MM with 10 mM succinate and again incubated as before. For soil growth experiments, cells were recovered from exponentially growing cultures on succinate (at culture turbidity OD_600_ = 0.4) by centrifugation (4000×*g* for 5 min at room temperature), resuspended in MM without further carbon source and then transferred to the soil microcosms (see below). Growth of *P. veronii* was determined by CFU counting of appropriate dilutions on MM plates with toluene (gas phase). *P. veronii* colonies were differentiated from any background soil bacterial colonies growing on MM plates with toluene by their green fluorescence, and counted under a digital dual band microscope (Dino-Lite model AM4115T-GRFBY) using the 485 nm blue LED excitation and 510 nm emission filter.

### Soil types and contaminated material

Three natural soils were used for microcosm growth studies with *P. veronii*. These consisted of (i) a sandy soil sampled in a lake Geneva beach near in St. Sulpice – named *Sand*, (ii) a silty soil sampled from the bank of the local stream ‘Sorge’ on the university campus – named *Silt*, and (iii) a clay soil sampled in a forest area on campus near to Lake Geneva – named *Clay*. Quantities of ~ 5 kg were spread on aluminium foil and air-dried on the laboratory bench. Sand and Silt were dried for 7 days (further water losses were not evident with longer drying periods), whereas Clay was dried for 15 days. At the moment of microcosm inoculations, the Sand, Silt, and Clay had gravimetric water content (GWC) of 0.14 ± 0.01%, 0.20 ± 0.01%, and 2.22 ± 0.06%, respectively. pH-H_2_O of the materials was 7.14 ± 0.02 (Sand), 8.57 ± 0.2 (Silt) and 7.78 ± 0.02 (Clay). Total organic matter content amounted to 0.028 (Sand), 0.13% (Silt) and 4.0% (Clay). Total cell counts on washed material and stained by SYBR Green I were quantified according to Weinbauer et al. [26]. The effect of drying on viable bacteria was determined with freshly sampled Sand dried for 3 h and for 5 days at ambient air temperature (20 °C, Supplementary Figure 2).

The polluted material (Jonction) originated from a former gasification work in Geneva and was obtained through collaboration with Biotech S.A., Geneva (CH)*.* According to previous characterization [27], the material contained primarily alkanes (C_10_ – C_40_; 9500 mg kg^− 1^) and polycyclic aromatic hydrocarbons (2300 mg kg^− 1^). In addition, minor concentrations of benzene (3.3 mg kg^− 1^) and methylated monoaromatic compounds were found (22 mg kg^− 1^). The original material consisted mainly of gravel (1–5 cm) covered with a layer of tar-containing mud. In order to obtain more easily handleable material, it was mixed 65/35 (*w/w*) in small portions with air-dried Silt and sieved through 3–mm diameter to remove the gravel. In the main text we refer to this mixed and sieved material as *Jonction*. At the moment of inoculation, Jonction had a GWC of 6.55 ± 0.15%.

### Growth kinetics estimations

The kinetics of *P. veronii* miniTn*5::gfp* population growth in soil was assessed in microcosms artificially contaminated with toluene. Four replicate microcosms were prepared, each consisting of 95 g of dried Sand, Silt or Clay inside 500–ml glass Schott bottles and closed with Teflon-lined screw caps. As control for porosity effects, we used autoclaved quartz at 5% GWC (silica crystals, 50–70 mesh particle size, Sigma-Aldrich ref. 274,739); hereafter referred to as artificial porous matrix or APM.

Toluene was dosed through the vapor phase from a sealed 1–ml micropipette tip, placed inside the glass bottle and containing 0.2 ml of pure toluene. Tubes with toluene were removed before inoculation with 5 ml suspension of washed preculture of *P. veronii* to obtain 2.5 × 10^4^ CFU g^− 1^ material at the start. Microcosm flasks were mixed on a horizontal roller mixer (IKA roller 6 digital) at 80 rpm for 30 min, with manual shaking every 5 min to detach the soil from the walls. After mixing, the tip containing the toluene was placed back inside and the bottles were incubated upright at 24–26 °C in the dark for 60–120 h with regular sampling (see below).

Growth of *P. veronii* miniTn*5::gfp* in Jonction was followed in (triplicate) microcosms of 10 g in 50 ml polypropylene screw-cap tubes (Greiner AG, cat #227261) containing either 100% Jonction (see above), 100% Silt, or mixtures of 75% (*g g*^− 1^), 50, and 25% Jonction with Silt. Microcosms were again inoculated with washed *P. veronii* preculture (0.5 ml) to achieve 2.5 × 10^4^ CFU g^− 1^ at the start. Microcosms were homogenized and incubated as above, either without any further addition of carbon, or amended with a sealed 1 ml micropipette tip containing 20 μl pure toluene (as described above).

Growth of *P. veronii* on toluene in liquid suspended culture was measured from four replicate 100–mL screw-cap conical flasks containing 15 ml MM and starting cell concentrations of ~ 1 × 10^7^ CFU ml^− 1^. As we found in preliminary experiments that *P. veronii* growth on toluene in liquid culture was less consistent with gas-phase dosing, we deployed an inert oil-toluene mixture instead. For this, we mixed 1:25 (*v/v*) of toluene:tetradecane (≥99.9%; Sigma-Aldrich ref.: 34866; Aldrich ref.: 87140) and added 0.5 ml per 15 ml liquid culture. Flasks were incubated at 30 °C and at 180 rpm on an orbital shaker.

Soil and liquid microcosms were sampled directly after inoculation (1 h) and then four times per day at approximately 6 h intervals (or once, for the Jonction series). Samples of 5 g (or 2.5 g for Jonction) were retrieved from the microcosms with a spatula and transferred to clean 50 ml polypropylene tubes. 10 ml of sterile saline solution (0.9% NaCl) was added to each tube and cells were extracted by vortexing for 1 min. Larger soil particles were allowed to sediment for a few seconds, after which the supernatant was transferred to a fresh 50 ml tube and serially diluted in sterile saline. For liquid cultures, a 1–mL aliquot was taken directly from the flask and serially diluted. Serial dilutions were drop-plated (10 × 5 μl drops of each dilution) on MM agar, which was incubated with toluene vapor to quantify the number of *P. veronii* CFU as described above. Identities of *P. veronii* miniTn*5::gfp* colonies were verified by their GFP fluorescence.

Growth rates (μ_max_) of *P. veronii* were calculated from the slope of the mean log_10_ CFU g^− 1^ over time across quadruplicate assays (or triplicates, for Jonction incubations). We further refer to the maximum population size (max pop size) as the highest mean CFU g^− 1^ or ml^− 1^ observed during the entire experiment for each condition, averaged from four (soils) or three (Jonction) replicates at the same sampling time point. Growth rates were compared among treatments by ANOVA, followed by post-hoc Tukey testing.

### Genome-wide gene expression analysis

For genome-wide expression analysis we used *P. veronii* wild-type, inoculated in the different materials or conditions and sampled after 1 h (transition phase or LAG), during estimated exponential (EXPO) or in stationary (STAT) phase.

For the transition phase we inoculated 5 ml (2.5 ml for Jonction) of a suspension of ~ 10^7^ ml^− 1^ *P. veronii* cells into soil microcosms with 95 g material (47.5 g for Jonction material) in 500 ml capped-glass Schott bottles, with toluene dosage through the gas phase. Cells were prepared from exponentially growing liquid cultures as described above, but resuspended in MM with 0.5 mM succinate to avoid starvation [20]. We used a higher starting cell density for the transition phase than in the exponential and stationary phases described below, in order to be able to extract sufficient amounts of RNA. As materials we tested here Sand, Silt, Clay, Jonction, APM and liquid suspended growth (LQ). All treatments were started in quadruplicates and incubated at room temperature (24–26 °C) without agitation for 1 h, after which 10 g were sampled from each replicate.

**Fig. 1:**
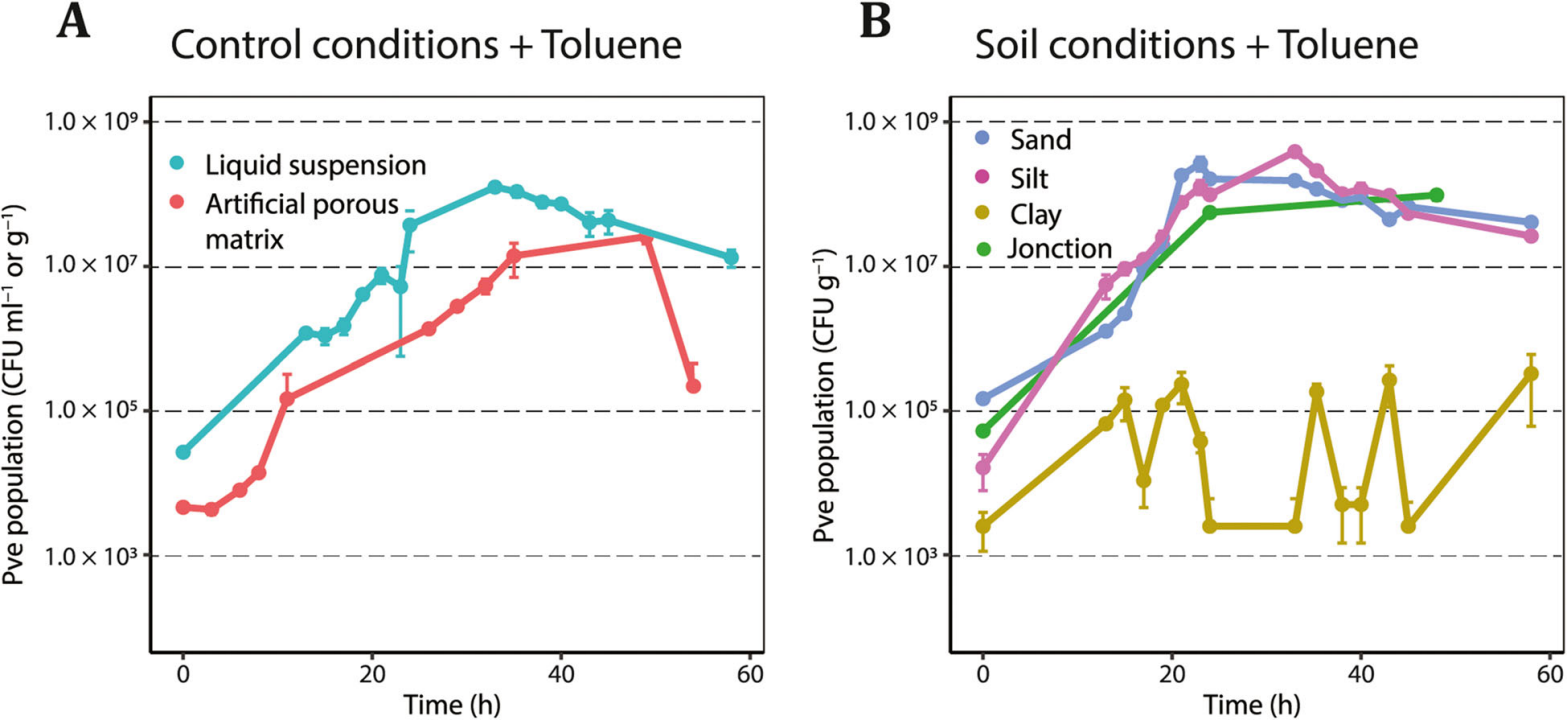
Effect of porous matrix on growth of *P. veronii* miniTn*5::gfp* with toluene. **a** *P. veronii* (Pve, strain 3381) growth in liquid suspension or artificial porous matrix. **b** Growth in contaminated (Jonction) and three natural non-sterile soils, in presence of externally added toluene. Data points show the mean number of colony forming units (CFU) ml^−1^ (for liquid suspension) or g^−1^ (for porous material) from independent biological triplicates, plotted on a logaritmic scale. Error bars represent calculated standard deviations from the mean. Identity of *P. veronii* colonies on MM plates with toluene vapor verified by their GFP fluorescence

*P. veronii* during exponential growth and stationary phase was sampled from similar inoculated microcosms (95 g material or 47.5 g for Jonction), but in this case with a low starting cell density of ~ 10^4^ *P. veronii* CFU g^− 1^ or ml^− 1^ to achieve sufficient growth in the soils. Cells were precultured as before, but washed and resuspended in MM without succinate. Microcosms were dosed with toluene as before (four replicates each), and sampled (10 g) at approximate exponential and stationary phase for *P. veronii* based on initial growth experiments as in Fig. 1. As materials we tested here Silt, Jonction and APM. For the liquid suspension control, *P. veronii* was inoculated into 100 ml screw-cap conical flasks containing 25 ml of MM amended with 0.5 ml of a 1:19 mixture of toluene:tetradecane to obtain a starting OD_600_ of 0.16 (four replicates). Flasks were incubated at 30 °C and 180 rpm on an orbital shaker. Cells were sampled after 4 h (OD = 0.5, 2 ml) and 24 h (OD = 1.8–1.9, 1 ml), and harvested by centrifugation at 3500×*g*, for 6 min at 30 °C. Pellets were snap-frozen in liquid nitrogen and stored at *−* 80 °C until RNA extraction.

In order to improve yields for RNA extraction and limit humic acid interference, we first washed cells from solid samples of 10 g by adding 20 ml of 4 °C-cold sterile saline solution (0.9% NaCl) to the tube (50 ml volume) and vortexing for 2 min. Suspensions were allowed to sediment for 1 min, after which the cleared liquid was transferred into a clean 50 ml tube, which was centrifuged for 4 min at 4000×g at 4 °C to collect the cells. After centrifugation, the supernatant was immediately discarded by inversion, the excess of liquid from the walls was quickly removed with a clean paper towel, and the cell pellet was snap-frozen in liquid nitrogen and stored at − 80 °C until RNA isolation. Estimated cell recoveries from the washing procedure are reported in Supplementary Figure 3.

### RNA isolation, purification and library sequencing

RNA was extracted from frozen cell samples using the RNA PowerSoil Total RNA Isolation Kit (MoBio Laboratories). Cells in thawed samples were disrupted in a bead-beating protocol as recommended by the manufacturer (MoBio Laboratories). RNA was purified following MoBio procedures and the final RNA pellet was resuspended in a volume of 20 μl of RNase-free water. Contaminating DNA was removed by two consecutive treatments with TURBO DNase (Ambion), followed by purification using an RNeasy MinElute Cleanup kit (QIAGEN, Valencia, CA, USA).

RNA quantity (reported in Supplementary Table 1) and quality was verified by reading the 260 nm absorbance and the absorbance ratios at 260/280 nm and 260/230 nm on a NanoDrop spectrophotometer (ThermoFisher Scientific). RNA was migrated on an Agilent 2100 Bioanalyser (Agilent Technologies) to verify the presence of intact 16S and 23S rRNA. Genomic DNA contamination was checked by PCR using specific primers for a unique region in the *P. veronii* genome. RNA samples were then depleted from ribosomal RNAs by using the Ribo-Zero rRNA Removal Kit Bacteria protocol (Epicentre, Madison, WI, USA). Subsequently, the RNA was reverse-transcribed, indexed and amplified by PCR using the ScriptSeq™ v2 Bacteria kit and ScriptSeq™ Index PCR primers set 1 (Epicentre). The resulting directional RNAseq libraries were sequenced using single 100-nt read chemistry on an Illumina HiSeq 2500 platform (Illumina, Inc., San Diego, USA) at the Lausanne Genomic Technologies Facility.

### Bioinformatic analysis and statistics

Raw reads were quality-filtered, mapped, sorted and indexed with Bowtie2 [28] and Samtools [29] under default settings, using the finalized gapless *P. veronii* genome sequence as reference (European Nucleotide Archive under bioproject number PRJEB11417). A summary of the total number of mapped reads per condition is listed in Supplementary Table 1. Mapped reads passing default alignment values were counted with HTSeq [30], then further processed and analysed with edgeR [31]. Only reads counted more than once per million in at least three replicates were kept. Counts were normalised across samples and compared between pairwise conditions in a modified Fisher’s exact test (as implemented in edgeR). ANOVA (also as implemented within edgeR) was used to detect differential gene expression with interpretation groups “natural soils – 1h” (Sand, Silt and Clay) and “controls – 1h” (Liquid and APM) or “polluted soil – 1h” (Jonction) and “controls – 1 h”. Comparative data of *P. veronii* transition response (1 h) in liquid suspension and in Sand were taken from previous work [20].

Genes were called significantly differentially expressed between two conditions at a false-discovery rate (FDR) of < 0.05, *p*-value of < 0.01 and a log_2_ fold-change > 2, and were subsequently interpreted by using Gene Ontology (GO) analysis [21]. GO terms of *P. veronii* genes were inferred using the program BLAST2GO [32]. The same program was then used to analyze for enrichment of significantly differentially expressed genes in pair-wise comparisons, using the Fisher’s exact test and correcting for multiple testing. A simplified network of common and soil-specific enriched GO terms for the biological process category was manually constructed in Cytoscape (version 3.7.2) [33] with nodes representing a biological process and edges the connecting parent-child.

A survey of *P. veronii* 1YdBTEX2 metabolic capacities was extracted from its reconstructed genome-scale metabolic model (iPsvr), which accounts for 1234 metabolic genes [23]. These genes translate potentially into 1812 metabolic reactions. Gene-reaction associations were extracted from the model, using the in-built RAVEN [34] functions, and linked to their potential corresponding metabolic pathways. iPsvr is curated based on KEGG [22] and all the reported reactions, metabolites and pathways follow KEGG nomenclature. Normalized gene expression values for the 1234 metabolic genes under the different conditions were calculated and compared using edgeR [31]. Identified KEGG reactions were visualized on a general metabolic map using iPath3 [35] with line thickness representing the log_2_ normalized expression attributed to that reaction. Those reactions whose mean expression differed by two or more standard deviations in EXPO phase among cells in liquid, Silt or Jonction, were highlighted in different color. Maps were exported to Adobe Illustrator (vs 2020). The mean normalized expression of all genes attributed to KEGG reactions under all conditions was further visualized as a heatmap with rows (i.e., genes) clustered in Euclidian distance (MATLAB vs 2016a, *clustergram* function).

### Database submission

The raw unmapped RNA-seq reads related to this study have been deposited in the NCBI Short Read Archive under Bioproject accession number PRJNA682712.

## Results

### Comparative growth of *P. veronii* in different soils with toluene as added carbon substrate

In order to benchmark growth of *P. veronii* in different soils, we inoculated microcosms dosed with toluene in comparison to liquid suspended medium or to artificial porous medium (APM, Fig. 1). Growth rates of *P. veronii* miniTn*5::gfp* with toluene in polluted (Jonction) and two natural soils (Sand and Silt) were slightly higher and statistically different from those measured in liquid or in APM (p_adj_ = 3.49 × 10^− 5^, ANOVA, followed by post-hoc Tukey test, Table 1). Maximum population sizes were highest in Silt and Sand, then liquid culture and Jonction material, followed by APM and Clay (Table 1). In liquid culture and in all microcosms, except perhaps in Jonction, the viable *P. veronii* population size decreased 8–10 fold after its maximum (Fig. 1), suggesting cell death. An even more pronounced decline was observed in APM (Fig. 1a).

**Table 1:** *Pseudomonas veronii* miniTn5::*gfp* growth kinetic parameters on toluene^a^ in porous media and in liquid suspension

Parameter^b^	Control		Natural soil			Polluted
	Liquid	APM^c^	Sand	Silt	Clay	Jonction^d^
max pop size	1.1 × 10^8^	2.6 × 10^7^	2.7 × 10^8^	3.9 × 10^8^	2.7 × 10^5^	1.4 × 10^8^
μ_max_	0.22 ± 0.01	0.23 ± 0.03	0.27 ± 0.01	0.32 ± 0.01	0.27 ± 0.4	0.29
G	2.43 ± 1.06	2.66 ± 0.03	2.60 ± 0.11	2.43 ± 0.26	2.51	2.39
N	10	10	11	13	5.6	10

^a^ Toluene dosage in liquid culture was dosed from a secondary oil-phase, whereas in soils and APM it was dosed through the gas phase

^b^max pop size = mean maximum population size (CFU g^− 1^ for solid matrix, and CFU ml^− 1^ for liquid suspension); μ_max_ = mean maximum growth rate ± stdev (h^− 1^, *n* = 3); G = generation time (h). N = estimated mean number of generations of growth

^c^*APM* Artificial porous matrix

^d^Single replicate because of insufficient material. Jonction here is 100%

In contrast to the other microcosms, the *P. veronii* miniTn*5::gfp* population in Clay developed much poorer, never reaching more than 2.7 × 10^5^ CFU g^− 1^ (Fig. 1b). Furthermore, the mean relative variability between replicates in Clay was higher (65% vs 8–38%), and CFU numbers increased only one order of magnitude in comparison with the measured inoculated *P. veronii* population size (1.28 × 10^4^ CFU g^− 1^). Population size variations in Clay suggested alternating growth and decline, perhaps as a result of cycles of predation (given that the soils were not sterilized). Predation may have been more pronounced in the Clay microcosms because of their slightly higher starting water content (Methods), in which protozoan may have remained alive for longer than in air-dried Sand or Silt [36–38]. Growth of native soil microbiota on added toluene was observed for Clay and Silt but not Sand (Supplementary Figure 4), suggesting that some substrate competition may have occurred with the inoculated strain.

### *P. veronii* growth in soil as a function of degree of contamination

**Fig. 2:**
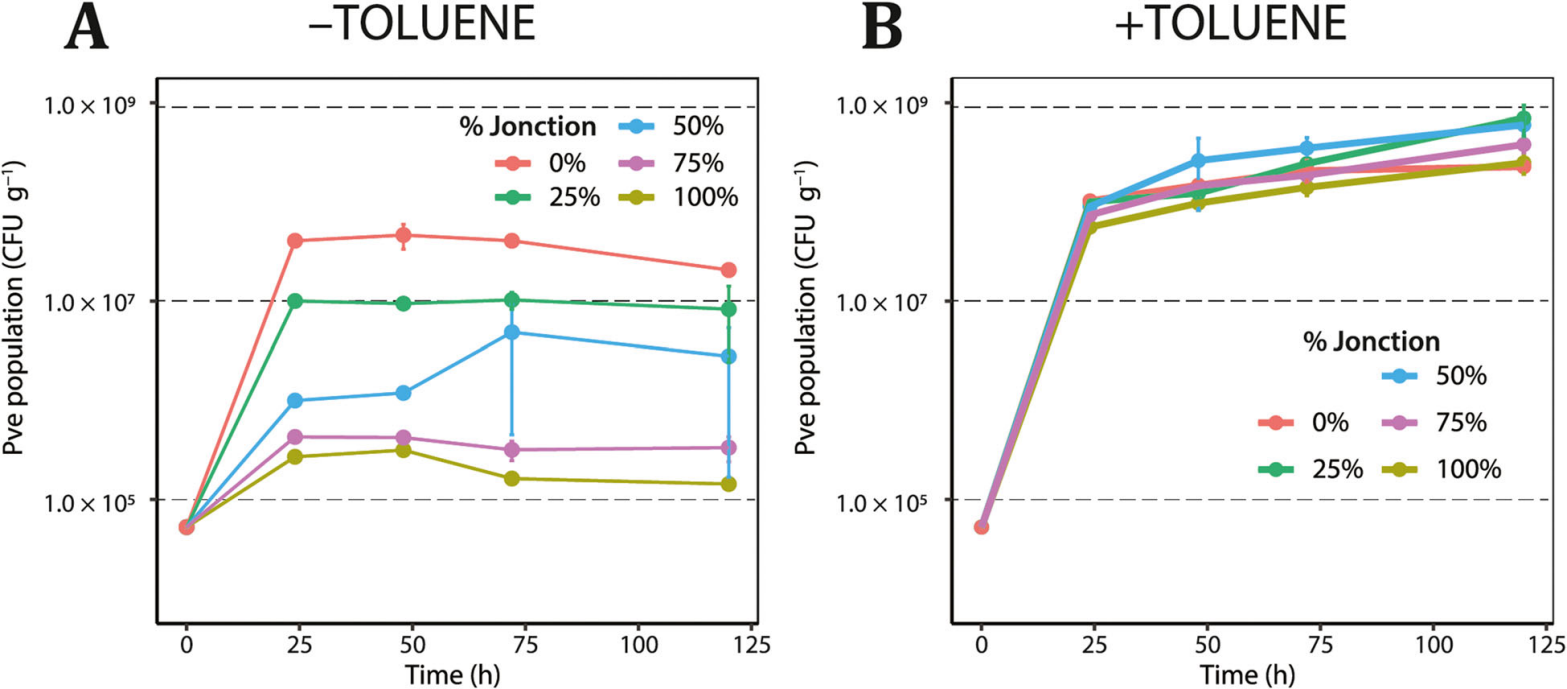
Growth of *P. veronii* miniTn*5::gfp* in soil as a function of different degree of contamination. **a** *P. veronii* (Pve, strain 3381) growth in material mixed from Silt and Jonction (colors correspond to percentage Jonction in *g/g* of total), without added toluene. **b** As **a**, but in presence of gaseous toluene. Data points are plotted on logaritmic scale and represent the means from independent biological triplicates with error bars showing calculated standard deviations. Identity of *P. veronii* colonies on MM plates with toluene vapor verified by their GFP fluorescence

Next, we tested growth in microcosms consisting of Silt-Jonction mixtures with different degree of contamination (Methods), in order to determine to what extent *P. veronii* could grow in field-collected polluted soil. Since contaminated material from Jonction contains monoaromatic compounds (see above) [27], we hypothesized that *P. veronii* might be able to grow in absence of externally added toluene. Contrary to our expectations, however, *P. veronii* miniTn*5::gfp* developed poorer in microcosms with a higher degree of Jonction material but without added toluene (Fig. 2). Whereas *P. veronii* grew to a population size of 4.1 × 10^7^ CFU g^− 1^ in Silt (without added toluene) within 24 h, in 100% Jonction it only reached 2.7 × 10^5^ CFU g^− 1^. Mixing Jonction material with Silt overproportionally reduced the final attained *P. veronii* population size (Fig. 2a). For example, with 50% Jonction:Silt the final *P. veronii* population size was only one-tenth from that on Silt alone. This indicated that some carbon is available for *P. veronii* growth in (non-sterile) Silt, but that the strain is inhibited by components or factors originating from Jonction. The poorer growth in microcosms mixed with Jonction material further suggested that *P. veronii* found little available aromatic substrates (Supplementary Figure 4).

In contrast, *P. veronii* grew very rapidly in all Jonction microcosms to which external toluene was supplied through the gas phase (Fig. 2b), and reached higher maximum population sizes than in those without (Fig. 2a), indicating that the cells were mainly using toluene as carbon source. Also native microbiota profited from addition of toluene (Supplementary Figure 4). There was less of an effect of increasing Jonction proportions, with highest population growth in the microcosms with 25% Jonction compared to higher Jonction proportions. Toluene may thus have given *P. veronii* some advantage to provide the energy necessary to protect itself from potentially harmful substances present in Jonction.

**Fig. 3:**
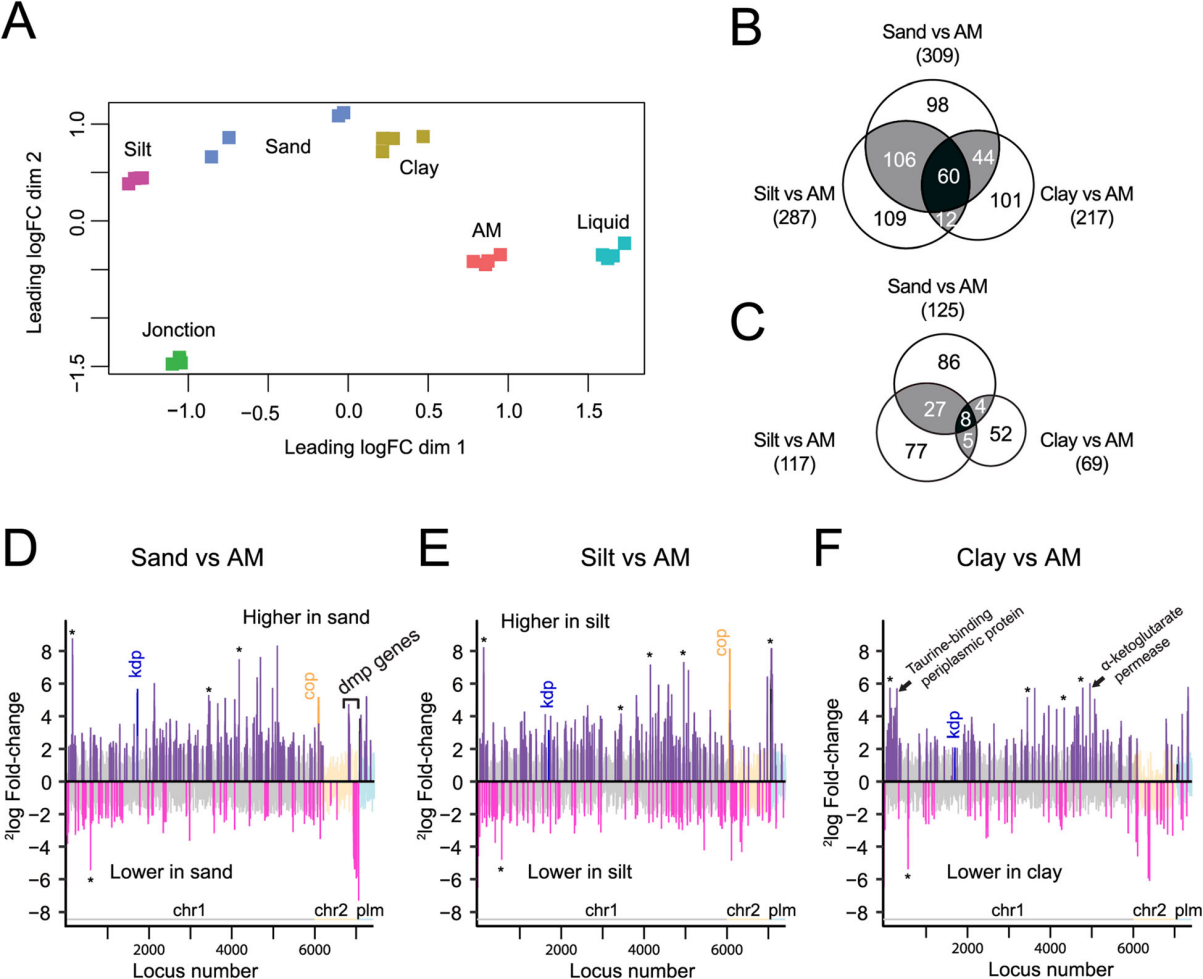
Genome-wide gene expression differences in wild-type *P. veronii* after 1 h exposure to different soils in presence of toluene. **a** Multi-dimensional scaling (MDS) plot of quadruplicate RNA-sequencing data sets for each of the soils, in comparison to liquid and artificial porous matrix (APM), and Jonction. Distances correspond to leading log-fold-changes between each pair of RNA samples. Leading log-fold-change is the average (root-mean-square) of the largest absolute log-fold changes between each pair of samples. **b** Statistically significantly higher expressed genes (i.e., those with log_2_-fold-change > 2, false-discovery rate < 0.05, *P* < 0.01) in each soil vs. APM. **c** as **b**, for lower expressed genes in soil than APM. **d**–**f** Expression profiles of the comparisons between sand, silt and clay vs APM, respectively, plotted as function of genome location (chr1, chromosome 1, grey background bars; chr2, chromosome 2, yellow; plm, plasmid, blue). Bars organized according to the locus_tag number. Bars indicate the mean log_2_-fold change. Dark purple, statistically significantly higher; pink, lower expressed genes. Asterisks point to a number of gene clusters common to all three soil-APM comparisons. *Kdp, cop* and *dmp* gene clusters; see description in main text

We subsequently used this physiological context to measure the genome-wide expression differences of wild-type *P. veronii* as a function of material and growth phase. We differentiated and compared three growth phases across the different materials: a transition phase (1 h after inoculation), exponential growth and stationary phase (time points depending on material and estimated from growth of the GFP-tagged variant, as defined in Table 2). Global transcriptomic responses of *P. veronii* to the different materials and conditions were very consistent, given distinct and good replica clustering in multi-dimensional scaling (Fig. 3a).

**Table 2:** Comparison groups and conditions of genome-wide gene expression analysis

Phase	Sample^a^	Material	Sampling Time (h)^b^	Comparison groups
Transition	LQ-1H	Liquid	1	
	APM-1H	Pure silica	1	APM-1H/LQ-1H
	SILT-1H	Silt	1	SILT-1H/APM-1H
	SAND-1H	Sand	1	SAND-1H/APM-1H
	CLAY-1H	Clay	1	CLAY-1H/APM-1H
	JN-1H	Jonction	1	JN-1H/APM-1H JN-1H/soils-1H
Exponential	LQ-EXPO	Liquid	4	LQ-EXPO/LQ-1H
	SILT-EXPO	Silt	29	SILT-EXPO/SILT-1H
	JN-EXPO	Jonction	18	JN-EXPO/JN-1H
Stationary	LQ-STAT	Liquid	24	LQ-STAT/LQ-1H
	APM-STAT	Pure silica	70	APM-STAT/APM-1H
	SILT-STAT	Silt	72	SILT-STAT/SILT-1H

^a^*LQ*, liquid suspension; *JN*, jonction; *APM*, artificial matrix; *1H*, transient 1-h exposure; *EXPO*, exponential growth phase; *STAT*, stationary phase

^b^Sampling time corresponds to Fig. 1a and b

### Effect of porosity on the transcriptomic transition response of *P. veronii*

In order to distinguish potential effects originating from matrix porosity, we first compared transcriptomes from inoculated *P. veronii* after 1 h in APM microcosms and in liquid suspension (LQ), both in presence of toluene (Table 2). In comparison to LQ, a total of 319 *P. veronii* genes were differentially expressed in APM (131 higher, 188 lower; Supplementary Data 1). One-third of the APM-higher expressed genes coded for conserved or secreted hypothetical proteins. GO analysis showed no particular enrichment of any biological function or pathway (Supplementary Data 2). However, APM-exposed cells showed 4–5 fold increased expression of a gene cluster for the uptake (*potGHI)* and metabolism (*puuACD,* PVE_r1g3330–3336) of putrescine (Supplementary Table 2), which is an important molecule during carbon and nitrogen starvation and in stress defense regulation [39]. Furthermore, a glycine utilization system encoded by the *gcvH2-gcvP2-sdaA-gcvT2* gene cluster was also 4–6 fold higher expressed in APM (Supplementary Table 2).

Among the 188 lower expressed genes in APM compared to LQ, GO analysis indicated enrichment of biological processes involved in growth and energy generation (Supplementary Data 2), many of which were directly implicated in toluene metabolism (e.g., PVE_r2g0711–0717 [*dmpBCDEFGH*], PVE_r2g0739–0742 [*ipbAaAbAcAd*]; Supplementary Table 2). This suggested that *P. veronii* detected less toluene in APM than in liquid. Further genes with lower expression in APM included the *leuBCD* and the *ipdVbkdBbkdA2A1* operons involved in the synthesis of branched-chain amino acids such as leucine, isoleucine, and valine (Supplementary Table 2). GO analysis suggested higher oxygen availability to cells in APM, judged from lowered expression of 7 out of 13 genes from the NADH dehydrogenase complex I (*nuoHIJKLMN*), genes from the *nar* operon for nitrate respiration (*narGJKLY*), a gene encoding oxygen-independent coproporphyrinogen-III oxidase (*hemN*), and genes coding for the cbb3 cytochrome C oxidase isoforms, *ccoN1*, *ccoO* and *ccoG2* (Supplementary Table 2).

Collectively, these results indicated that cells in APM perceived carbon and nitrogen limitation, possibly differences in oxygen provision, triggering a response to start recycling nitrogen-rich compounds and amino acids, and decreasing the synthesis of branched-chain amino acids (i.e., leucine, isoleucine and valine). These effects may thus be solely a response to change in porosity from the growth matrix itself.

### Effect of soil types on the immediate *P. veronii* response

**Fig. 4:**
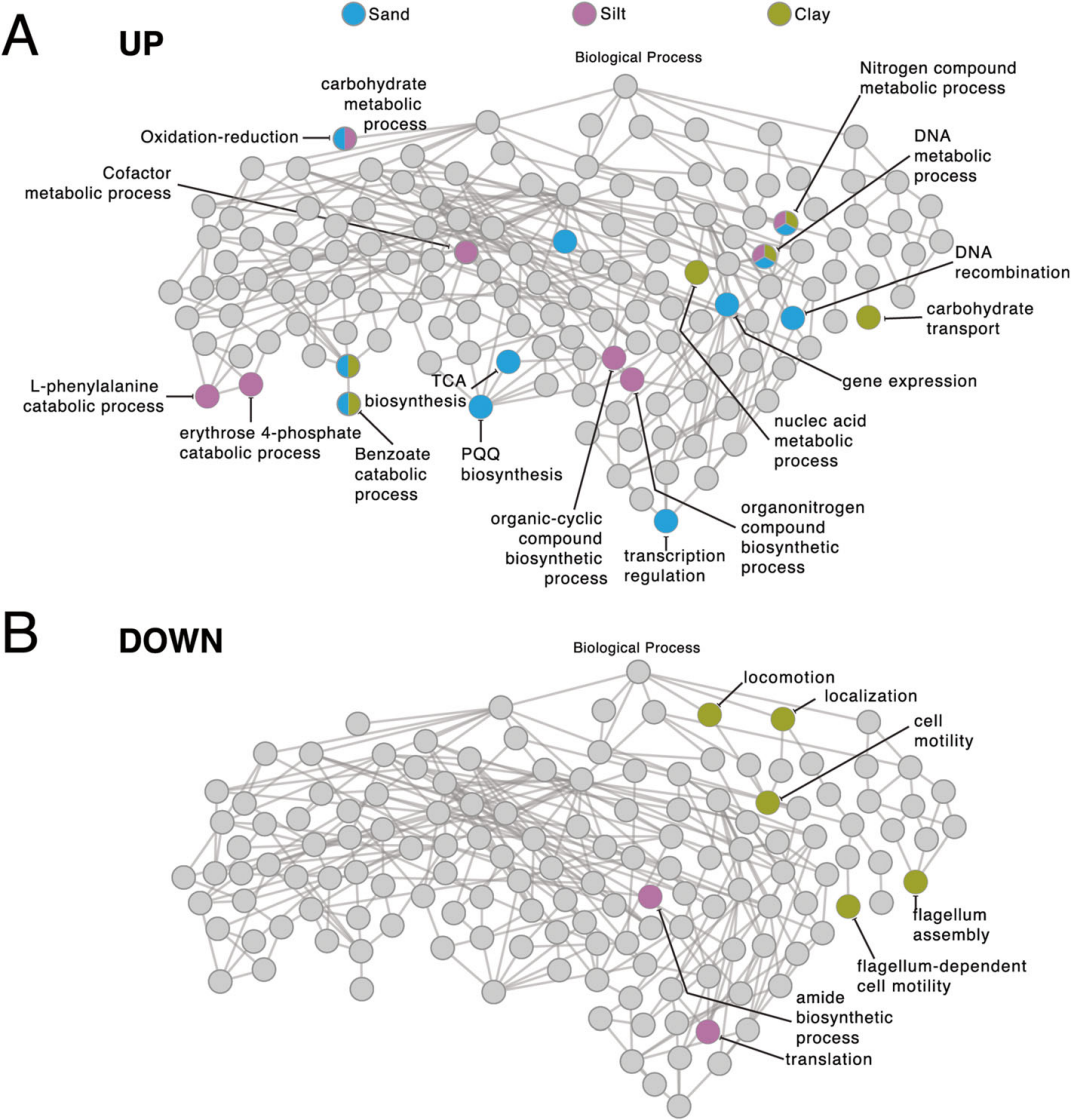
Simplified network of common and soil-specific enriched GO terms during 1 h transition. Colored circles represent GO terms specifically enriched in genome-wide gene expression of wild-type *P. veronii* under the hierarchy Biological process, in comparison to APM, for each of the three soils or in common, according to the legend

In comparison to APM, the transient contact of *P. veronii* in different soil types (i.e., 1 h Sand, Silt and Clay) caused a further common core of 68 genes (60 higher and 8 lower) to change expression (Fig. 3b and c, black zones, Table 3). Differences in gene expression were not located to specific genome positions or replicons (Fig. 3d–f). Commonly higher expressed biological processes in soils included, notably, the pathways “nitrogen compound metabolic process” (GO:0006807) and “DNA metabolic process” (GO:0006259), as well as “benzoate catabolic process”, “carbohydrate metabolism and transport”, and “aromatic amino acid catabolic process” (Fig. 4a, Supplementary Datas 3, 4, 5, 6, 7, 8, 9 and 10).

**Table 3:** Common differentially expressed genes of *Pseudomonas veronii* wild-type during transition in soils

Gene ID	Gene	Gene function	Log_2_ fold-change in comparison^a^					
			Sand vs APM	Silt vs APM	Clay vs APM	Jonction vs APM	One-way ANOVA^b^	
							Natural soils vs Controls	Jonction vs Controls
PVE_r1g2130	*lpdV*	dihydrolipoyl dehydrogenase	2.07	2.42	0.31	2.14	2.00	2.00
PVE_r1g2131	*bkdB*	dihydrolipoamide branched chain transacylase	3.07	2.39	1.75	3.11	2.57	2.00
PVE_r1g2132	*bkdA2*	2-oxoisovalerate dehydrogenase subunit beta	3.65	3.16	2.50	3.78	3.25	3.58
PVE_r1g2133	*bkdA1*	2-oxoisovalerate dehydrogenase subunit alpha	3.44	3.13	1.60	3.84	3.01	3.58
PVE_r1g5053	*hibA*	probable 3-hydroxyisobutyrate dehydrogenase	2.83	3.40	1.86	3.10	2.91	2.92
PVE_r1g5054	*ALDH6B2*	methylmalonate-semialdehyde dehydrogenase	3.63	3.54	2.91	3.98	3.45	3.81
PVE_r1g2545	*narK*	nitrate/nitrite transporter NarK	3.80	1.97	0.00	4.20	2.76	3.87
PVE_r1g2546	*narG*	respiratory nitrate reductase 1 alpha chain	3.72	2.33	−0.60	3.68	2.77	3.37
PVE_r1g2547	*narY*	respiratory nitrate reductase 2 beta chain	3.51	2.41	0.03	3.35	2.68	3.04
PVE_r1g2548	*narJ*	nitrate reductase molybdenum cofactor assembly chaperone	2.84	2.58	0.19	2.85	2.36	2.56
PVE_r1g2549	*narI*	respiratory nitrate reductase 1 gamma chain	2.60	2.14	0.11	2.56	2.04	2.29
PVE_r1g0681	*copA*	copper-exporting P-type ATPase A	2.39	3.30	0.38	3.51	2.56	3.22
PVE_r1g6093	*copC*	copper resistance protein C	4.53	6.00	1.61	3.22	5.04	3.05
PVE_r2g0903	*copC*	copper resistance protein C	1.27	6.39	1.21	3.35	5.00	3.12
PVE_p0049	*copB*	copper resistance protein B	3.00	2.86	1.63	2.58	2.68	2.45
PVE_p0050		hypothetical secreted protein	3.19	3.64	2.11	3.19	3.18	3.03
PVE_p0051	*copA*	copper resistance protein A	3.02	3.50	1.38	2.25	2.97	2.13
PVE_p0052		hypothetical secreted protein	4.04	6.54	0.70	3.53	5.35	3.26
PVE_r1g0242		membrane protein	2.78	2.25	2.60	2.51	2.57	2.51
PVE_r1g0243	*pgaB*	poly-beta-1,6-N-acetyl-D-glucosamine N-deacetylase	2.57	1.88	2.34	2.57	2.09	2.52
PVE_r1g0244	*pgaC*	poly-beta-1,6-N-acetyl-D-glucosamine synthase	2.29	1.40	2.03	2.52	2.09	2.46
PVE_r1g0245	*pgaD*	biofilm PGA synthesis protein PgaD	2.72	1.66	2.11	2.56	2.07	2.46

^a^ Log_2_ fold-change in condition compared with control in soil (FDR < 0.05, *p* < 0.01)

^b.^One-way ANOVA (analysis of variance) in the condition “soil”; natural or polluted vs controls (LQ and APM)

Soil-specific responses in comparison to APM comprised some further 200–300 genes (Fig. 3b and c, white or grey zones, Supplementary Data 3, 4 and 5). Particularly in Sand, these consisted of higher expressed GO-pathways “regulatory and metabolic processes”, “RNA biosynthesis” and “DNA-dependent transcription” (Supplementary Data 6). In Silt, the terms “translation” (GO: 0006412), “peptide biosynthetic process” (GO:0043043), and “amide biosynthetic process” (GO:0043604) were underrepresented (Supplementary Data 7, 8). In contrast, for Clay, enriched GO terms associated with decreased “cell motility” (GO: 0048870), “localization” (GO: 0051179), and “flagellum assembly” (GO:0044780, Fig. 4b, Supplementary Data 9, 10).

In comparison to both LQ and APM controls, there were 293 common genes at least four-fold higher and 22 four-fold lower expressed in soils (*p* < 0.05, ANOVA, Supplementary Data 11). About 29% of those encode either hypothetical proteins, hypothetical secreted proteins, membrane proteins, or conserved hypothetical proteins. The gene coding for ornithine aminotransferase (PVE_r1g5099) was among those whose expression increased the most in soils (165-fold), emphasizing the apparent importance for polyamine biosynthesis [39]. A number of genes with hypothetical function were induced at least by 100-fold (Supplementary Table 3). Other genes with significantly increased expression in soils included the *pgaABCD* operon, which is involved in the synthesis of extracellular polysaccharide. Also *tauAB*, *kdpFABC*, and at least two *copRSABCD* gene clusters, which are involved in taurine, potassium and copper transport, respectively, were upregulated in soil. Furthermore, gene clusters (PVE_r1g5622–5628) involved in malonate transport and metabolism, and of *bkdA1A2B*, involved in the metabolism of branched-chain amino acids, were higher expressed (Supplementary Table 3). The *dmpBCDEFGH* gene cluster for phenol and catechol *meta-*cleavage degradation together with two additional genes (PVE_p0191–0193) and the *narGIJKY* gene cluster for nitrate respiration were also significantly induced in *P. veronii* in soils after 1 h (Supplementary Table 3).

Collectively, these results thus suggested that, in contrast to APM*, P. veronii* cells in soils do not face nutrient limitation, and possibly gain additional proton motive force from nitrate reduction. Cells were adjusting their metabolism and transport systems for the available resources. The soil environment further triggered defense mechanisms to protect against osmotic stress or favor biofilm formation.

### Transition transcriptomic response of *P. veronii* upon inoculation into Jonction polluted material in the presence of toluene

**Fig. 5:**
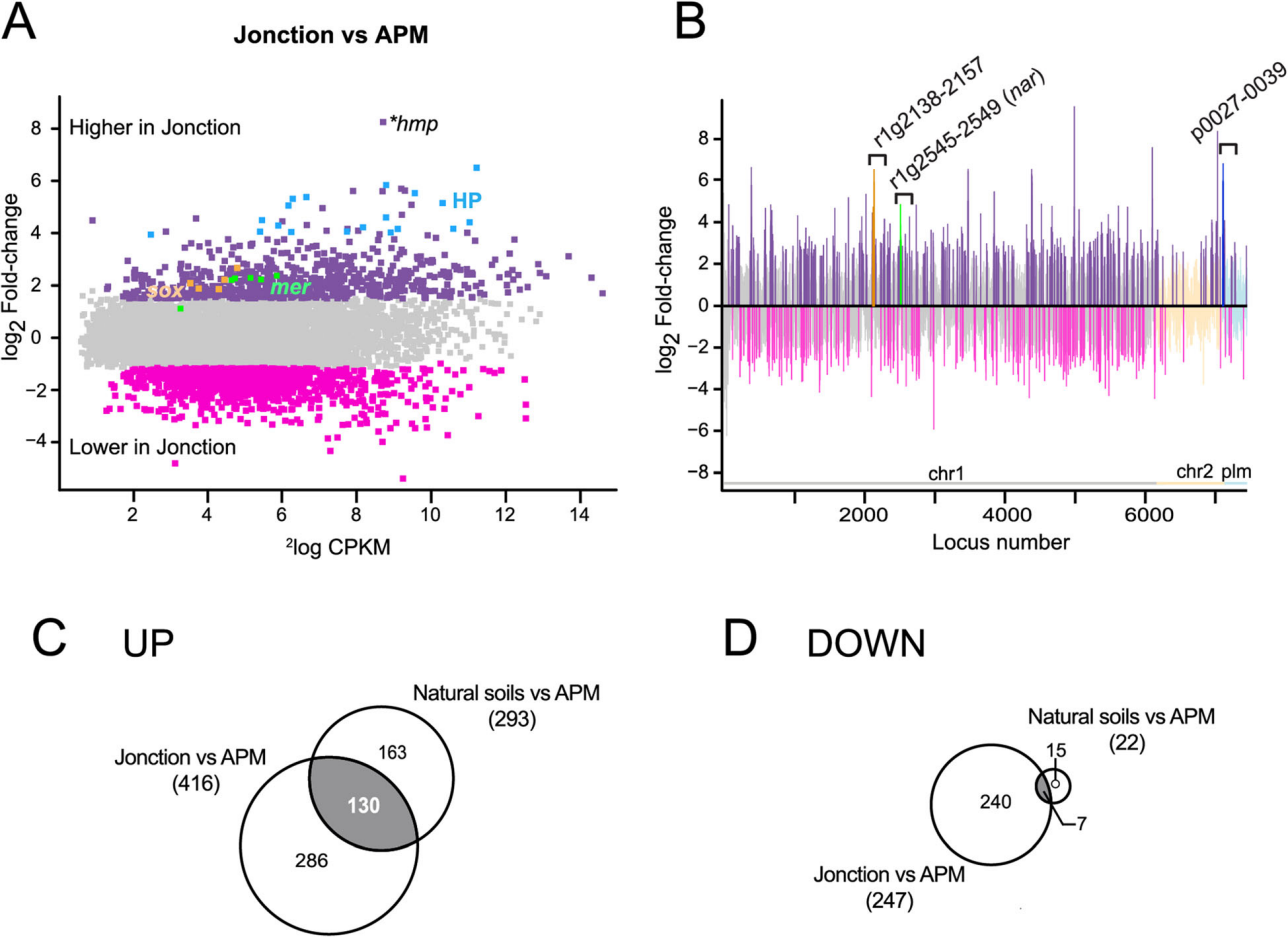
Genome-wide response of wild-type *P. veronii* after 1 h contact to Jonction material. **a** Global gene expression levels (log_2_ CPKM, counts per kilobasepair per million) versus their log_2_-fold change (FC), compared between cells in Jonction versus APM; in grey, genes not statistically differentially expressed (log_2_ FC < 1, False Discovery Rate [FDR] > 0.05, *P* > 0.01); magenta, genes with lower, and dark purple, genes with higher expression in Jonction. Blue, hypothetical proteins (HP) with log_2_ FC > 4.00; green, *mer* genes (mercury detoxification); yellow, *sox* genes (sarcosine metabolism). Asterisks point to the *hmp* gene; see main text. **b** Gene expression differences as per locus position and replicon (description similar to legend of Fig. 3d). **c** and **d** Numbers of unique (white zones) and common genes (grey zones) significantly higher (**c**, UP) or lower (**d**, DOWN) expressed between Jonction and APM, or soils and APM (at log_2_ FC > 2, FDR < 0.05, *P* < 0.01)

In order to understand whether adaptation of *P. veronii* to a field-polluted material is different than to artificially contaminated soils, we compared the transient response in Jonction material in the presence of toluene (Tables 3 & 4). Multi-dimensional scaling analysis confirmed that the transcriptomic response to Jonction was globally different from the clean soils, despite the common presence of externally added toluene (Fig. 3a). In comparison to APM alone (Fig. 5a), 416 genes were higher, and 246 lower expressed in Jonction (Fig. 5b, Supplementary Data 12). Approximately 30% of those correspond to (conserved) hypothetical and hypothetical secreted proteins, and (conserved hypothetical) membrane proteins. A total of 137 of Jonction-differentially expressed genes (130 up and 7 down) were shared with those of *P. veronii* in clean soils (Fig. 5c and d, Supplementary Data 13). These included genes involved in L-valine degradation, dissimilatory nitrate reduction, and copper transport (Table 3). Further common to clean and polluted soils was a strong induction of the *hmp* gene, coding for a flavohemoprotein (Fig. 5a and Supplementary Data 13), which has been described to be important in NO detoxification in response to nitrosative stress [40]. This suggests that cells are experiencing stress from noxious nitrogen compounds.

**Table 4:** Subset of Jonction-specific differentially expressed genes of *Pseudomonas veronii* wild-type during 1 h transition

Locus_tag	log_**2**_ FC^**a**^	Gene	Gene function
PVE_r1g0226	2.84		tail protein
PVE_r1g0616	211	*psiF*	Phosphate starvation-inducible protein PsiF
PVE_r1g0858	2.46	*clpB*	chaperone protein ClpB
PVE_r1g1263	2.17		tail protein
PVE_r1g1264	2.36		phage tail protein
PVE_r1g1271	2.36		pyocin R, lytic enzyme
PVE_r1g1633	2.23		universal stress protein A
PVE_r1g2181	2.10		RND transporter
PVE_r1g2307	3.59		multidrug RND transporter
PVE_r1g2308	3.57		multidrug transporter
PVE_r1g2309	2.29	*emrB*	multidrug export protein EmrB
PVE_r1g2514	2.34	*nirG*	Protein NirG
PVE_r1g2515	2.12	*nirL*	Protein NirL
PVE_r1g2516	2.05	*nirD*	Protein NirD
PVE_r1g2551	2.75	*moaA1*	cyclic pyranopterin monophosphate synthase 1
PVE_r1g2614	2.96	*moaB*	molybdenum cofactor biosynthesis protein B
PVE_r1g2615	2.41	*moaA*	molybdenum cofactor biosynthesis protein A
PVE_r1g2895	2.65		Transposase for insertion sequence element IS1328
PVE_r1g3164	2.33		universal stress protein
PVE_r1g3188	2.26		universal stress protein
PVE_r1g3381	2.43		RND transporter MFP subunit
PVE_r1g3768	2.26		universal stress protein
PVE_r1g4143	2.30	*htpG*	chaperone protein HtpG
PVE_r1g4506	2.17		nitrate reductase
PVE_r1g4749	2.36	*arcA*	arginine deiminase
PVE_r1g4750	2.77	*arcB*	ornithine carbamoyltransferase, catabolic
PVE_r1g4751	2.28	*arcC*	carbamate kinase
PVE_r1g5119	2.63	*dnaK*	chaperone protein DnaK
PVE_r1g5393	2.99	*cat*	catalase
PVE_r1g5544	2.13	*soxG*	sarcosine oxidase subunit gamma
PVE_r1g5545	1.92	*soxA*	Sarcosine oxidase subunit alpha
PVE_r1g5546	2.23	*soxD*	sarcosine oxidase subunit delta
PVE_r1g5547	2.70	*soxB*	Sarcosine oxidase subunit beta
PVE_r2g0272	2.51		Insertion element IS2A uncharacterized 48.2 kDa protein
PVE_r2g0273	2.07	*insC1*	transposase InsC for insertion element IS2
PVE_r2g0743	2.88	*insH5*	transposase InsH for insertion sequence element IS5Y
PVE_r2g0813	0.22	*merR*	mercuric resistance operon regulatory protein
PVE_r2g0814	2.30	*merT*	mercuric transport protein
PVE_r2g0815	2.24	*merP*	mercury resistance system
PVE_r2g0816	2.39	*merC*	putative mercury transport protein MerC
PVE_r2g0817	2.25	*merA*	mercuric reductase
PVE_r2g0818	2.29		conserved hypothetical membrane protein
PVE_r2g0819	2.13	*merD*	mercuric resistance transcriptional repressor
PVE_r2g0831	3.43	*insH5*	transposase InsH for insertion sequence element IS5Y
PVE_r2g0931	7.23	*tnpA1*	putative transposase, TnpA1
PVE_p0038	2.16		RND transporter
PVE_p0170	2.00		mobile_element
PVE_p0207	2.61		integrase

^a^In comparison to 1 h in APM

Among the genes induced specifically in Jonction and not in clean soils (286 genes, Supplementary Data 14), were those of the *moaA1BA* (PVE_r1g2551–2517) operon, which is responsible for molybdopterin biosynthesis (Table 4), and the *arcDABC* genes, responsible for L-arginine degradation. Furthermore, the *soxGADB-glyA3* cluster was induced in Jonction, which is responsible for the conversion of sarcosine into serine, and in *Pseudomonas aeruginosa* is involved in adaptation to using alternative carbon, nitrogen and energy sources for growth [41, 42]. In addition to the *nar* genes*,* some of the *nir* genes (*nirGLD*-PVE_r1g2514–2516) were also higher expressed in cells in Jonction, suggesting further induction of anoxic respiration pathways.

Finally, several other defense systems were higher expressed in Jonction (Table 4). These encompassed the *merTPCA-PVE_r1g0818* operon, which encodes for mercury resistance, genes coding for “Universal stress protein” [43], for chaperones such as *clpB*, *htpG*, *groS*, *groL* and *dnaK*, catalases (e.g., PVE_r1g0164 and PVE_r1g5393) and for the phosphate starvation-inducible protein PsiF. The GO enrichment analysis of transcriptomic response in Jonction was coherent with these observations (Supplementary Data 15, 16). This indicated that *P. veronii* perceived a more stressful environment in Jonction than in the clean soils, although all contained artificially added toluene.

### Genome-wide expression differences during growth

After having uncovered the specific expression differences during the transition of *P. veronii* into clean or contaminated soils compared to liquid and APM, we next focused on measuring its metabolic reprogramming during actual growth in soil. Given that the three soils had shown comparable global transition reactions (Fig. 3) and that *P. veronii* did not grow very well in Clay (Fig. 1), we restricted ourselves in this analysis to Silt. Unfortunately, we could not recover sufficient RNA for analysis from *P. veronii* growing exponentially in APM, nor from stationary phase cells in Jonction. Thus, we could finally compare six experimental conditions, all for cells grown in the presence of toluene (Table 2, EXPO and STAT phase).

**Fig. 6:**
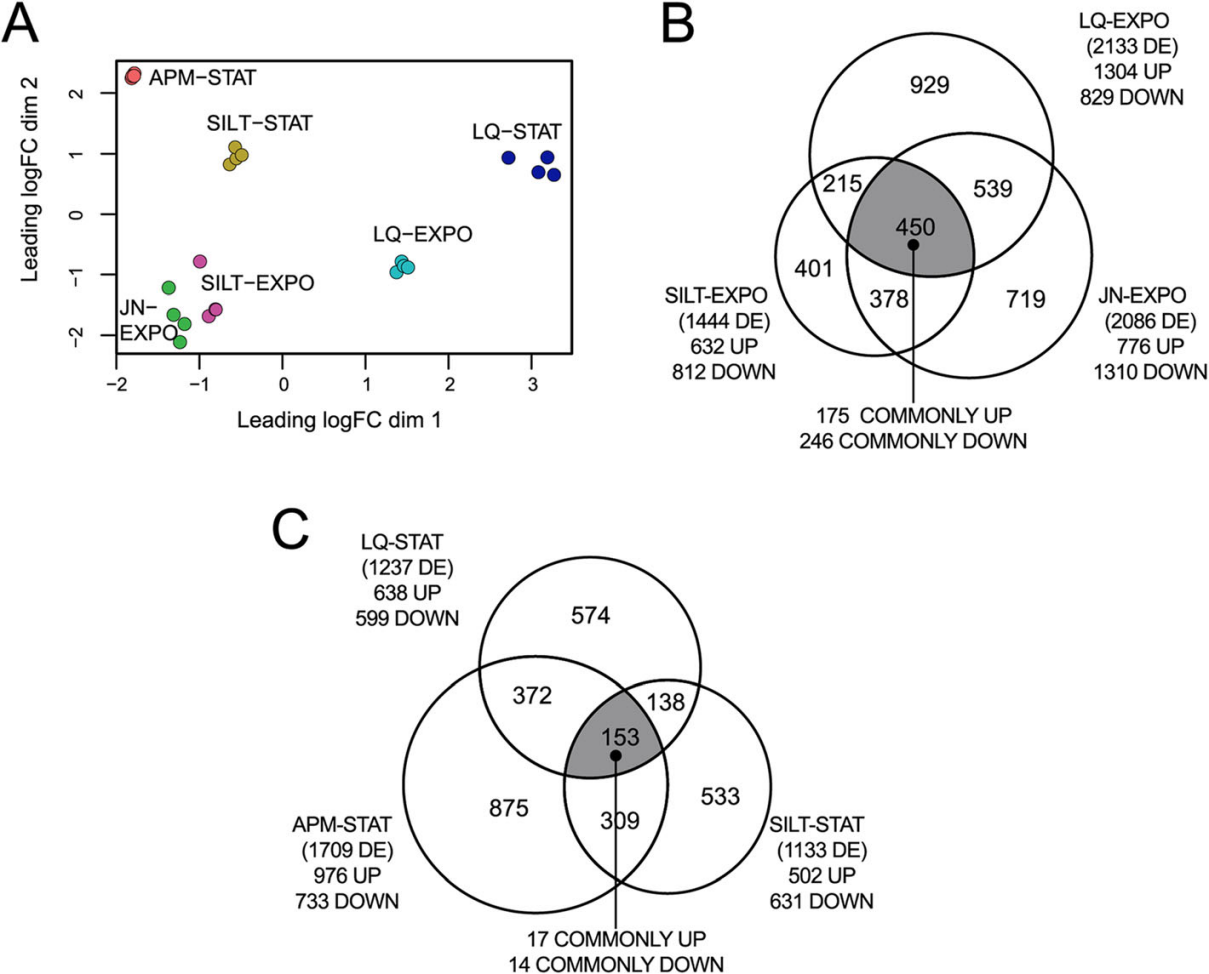
Global analysis of gene expression in exponentially growing and stationary phase cells of wild-type *P. veronii* in different materials. **a** Two-dimensional Principal Component Analysis of *P. veronii* transcriptome differences at the indicated growth phases and conditions compared to their respective 1-h expression (as specified in Table 2). See further legend to Fig. 3a. **b** Numbers of total, specific and common differentially expressed (DE) genes in pair-wise comparisons of *P. veronii* in exponential phase vs 1 h, or **c** in stationary phase vs 1 h. Dark shaded areas indicate the total number of *commonly* differentially expressed genes in the comparison groups. The difference of this sum to the total points to shared genes whose direction of expression is dissimilar (e.g., UP in one and DOWN in the other comparison)

Growth environments produced clear distinct global signatures with excellent replicate clustering, and EXPO was well-separated from STAT phase responses (Fig. 6a). A total of 175 genes were commonly higher and 246 lower expressed in exponential phase conditions compared to the 1 h transition (Fig. 6b, Supplementary Data 17), and 17 genes were commonly higher and 14 lower expressed in stationary phase compared to the 1 h signatures (Fig. 6c). The number of uniquely differentially expressed genes in any of the conditions surpassed those of the commonalities (Fig. 6b, c).

As expected, both GO analysis and individual gene annotations confirmed most of the commonly higher expressed genes in exponentially growing cells to be related to growth and energy production (Table 5, Supplementary Data 18, 19, 20, 21, 22 and 23). For example, higher expressed genes included those for (i) ribosome assembly and protein synthesis (*rpoAB, rps* and *rpl*), elongation factors (EF), *fusA* (EF-G), *efp* (EF-P), *tsf* (EF-Ts), and *tuf* (EF-Tu); (ii) DNA replication; (iii) ATP synthesis; (iv) sulfate transport; (v) TCA cycle, and (vi) amino acid biosynthesis.

**Table 5:** Common and specific biological processes enriched during exponential growth of wild-type *P. veronii*^a^

Regulation	Biological process	Liquid	Silt	Jonction
UP	ATP synthesis	*nuoEG, atpAFGH*	*nuoEGKMNL, atpAFGH*	*nuoEGKLMN, atpABCFGH*
	Aerobic respiration	*cyoABC*	*cyoABC*	*cyoABC*
	Toluene catabolic process		*dmpBCDFH, nahHIJKLMNOT*	*dmpBCDGH*
	Regulation of transcription		*tuf, lepA, efp, arnF, nusA, mfd*	*nusABG, rho, rpoD, rstA, gntR*
	Tricarboxylic acid cycle	*sucABC, sdhAB, acnB, icd*	*sucABC, sdhAB, acnB, icd*	*sucABC, sdhAB*
	Urea catabolism and transport		*ureABC*, urea ABC transporter	urea ABC transporter
	Translation-ribosome biosynthesis	*rpl, rps, prfABC*	*rpl*, *rps*	*rpl, rps*, *prfABC,* GTPase Der*,* GTPase Era
	Translation-elongation factor	*tsf, tuf, fusA, efp*	*tsf, tuf, fusA, efp*	*tsf, tuf, fusA, efp*
	Glutamate biosynthesis	*gltB, gltI*	*arnF, gltD, gltI*	
	Sulfate and taurine transport	*cysADW*	*cysADW, tauABC*	*cysADW*
	Protein folding	*surA, cyp18,*	*htpG, surA, groL,dnaJ, arnF, hscA, cyp18, ppiA*	
	Fatty acid biosynthesis	Malonyl-CoA - *accABCD*, *fabDF*		Malonyl-CoA - *accABCD*, *fabADFGZ*
	Water-soluble vitamin metabolic process	Inosine-*purADHLMTU*		Riboflavin - *ribDFH2*; thiamine - *thiIL; c*obalamine – *cobNPS*; f*olate - folDE2KD;* inosine *- purADEHKLMTU*
	Bacterial membrane organization	Peptidoglycan/LPS – *rfaCG;* cell shape determination – *minE, minD; Lipid A – lpxAB;* Isoprenoids - *ispEFG*; cell division *zapE*	Flagellum assembly – *fliDKM, flgBCDE, flaG*	Peptidoglycan/LPS - *uppSP, lptD, rfaPCG* Cell shape determination - *minE, mrdB, mreB, minD;* Lipid A – *lpxABDH*; Isoprenoids – *ispEFG;* cell division *zapE;* ferripyoverdine receptor *– fpvA*
	Transcriptional regulators:	AraC; ArsR	AraC; ArsR; MerR; LysR; TetR; LuxR; *ttgVW*	AraC; ArsR*, ttgRVW; qseB; norR; copG;*
DOWN	Organic substance metabolism	branched-chain amino acid - *bkdA1A2B*	branched-chain amino acid - *bkdA1A2B;* arginine *- arcABCD*; malonate - *mdcBCG*; biopolymer - *phbCB*; phenylalanine - *hmgA*, *maiA*; molybdenum - *moaAA1B*	branched-chain amino acid - *bkdA1A2BIpdV;* vanillate - *vanAB*; biopolymer -*phhAB*; arginine - *dauAB*;, molybdenum -*moaAA1B*; putrescine - *putA, puuABC*
	Nitrate respiration	*narIJ, nirQS, nosZ*	*narGIJLY,* *nirQS*, *norRR2*	*narGJKL,* *nirQS*, *nosZ*
	Transposition		integrase, transposases, base plate protein	transposases, insertion element
	Aerobic respiration	cytochrome B559-B561-CBB3; *cydB*	cytochrome B559-B561-CBB3; *cydB*	cytochrome B559-B561-CBB3; *cydAB, ctaD*; hem biosynthesis - *cpo, hemN, hemH*; ferrodoxin, cytochrome c oxidase
	Response to stress	flavohemoprotein – *hmp;* heat shock protein, universal stress protein A	flavohemoprotein – *hmp;* heat shock protein; Universal stress protein A; cold shock protein	flavohemoprotein - *hmp*; osmotic stress – *cysG;* heat shock protein; virulence sensor *bvgAS;* universal stress protein A; Catalase –*katE*; cold shock protein
	Transport			putrescine – *potAGHI*; glycine betaine -*opuAA-AB*; magnesium - *mgtAB*; mercury – *merATP*; RND transporter; sulfate transporter; citrate transporter, MFS transporter

^a^Enrichment defined in comparison to the 1 h transition phase transcriptome signatures

In contrast, signatures of commonly lower expressed genes were globally less clear. Around 50% of those encoded hypothetical proteins (Table 5). Others included transcriptional factors from the AraC and LuxE, or from unknown families, suggesting fine-tuning of metabolism to the uptake of resources. Genes for Universal Stress Protein A (PVE_r1g0156) and cold shock protein (PVE_r1g3189) were commonly lower expressed (Table 5), possibly associated with a release of stress in dividing cells.

Genes specifically higher expressed in exponential phase in Silt and Jonction included those for proteins involved in phenol and catechol *meta-*cleavage metabolism (*dmp* gene cluster) and urea transport (Table 5). This suggested that cells in soils were increasing flux through the *meta-*cleavage pathway, possibly because of higher perceived toluene availability than in liquid.

Among the genes specifically higher expressed during exponential growth in Silt (Supplementary Data 24) were those for taurine transport, urea degradation*,* chaperones, pilus biogenesis, flagellum assembly, and chemotaxis (Table 5), suggesting cells to be actively moving around, limited by and scavenging for nutrients. Interestingly, cells in Silt further activated the alternative *nah* pathway for aromatic compound metabolism (Table 5), in addition to *dmp.* This indicated usage of other aromatic carbon sources present in Silt, since the *nah* genes are not induced by toluene [20]. These aromatic compounds may be a common fraction of soil organic matter [44], and this alternate gene expression would explain the observed background growth in Silt without added toluene (Fig. 2a).

Genes induced specifically in exponentially growing cells in Jonction covered multiple unspecified transporters (such as sugar and phosphate ABC transporters, MFS transporters, Supplementary Data 25), uptake of ferripyoverdine, and ribosome biogenesis GTPases. Interestingly, several genes for cell division, cell shape, and peptidoglycan biosynthesis were higher expressed, such as the rod shape-determining proteins MreB and RodA, and the penicillin binding protein (PVE_r1g5075, Table 5). Defense mechanisms continued to be higher expressed in Jonction-growing cells, such as fusaric acid resistance protein (PVE_r1g1039), the multidrug resistance protein MdtC, and a gene coding for a beta-lactamase (PVE_r1g1543, Table 5). Also induction of many transporters may be a sign for defense against toxic compounds.

### Analysis of core metabolic reactions

**Fig. 7:**
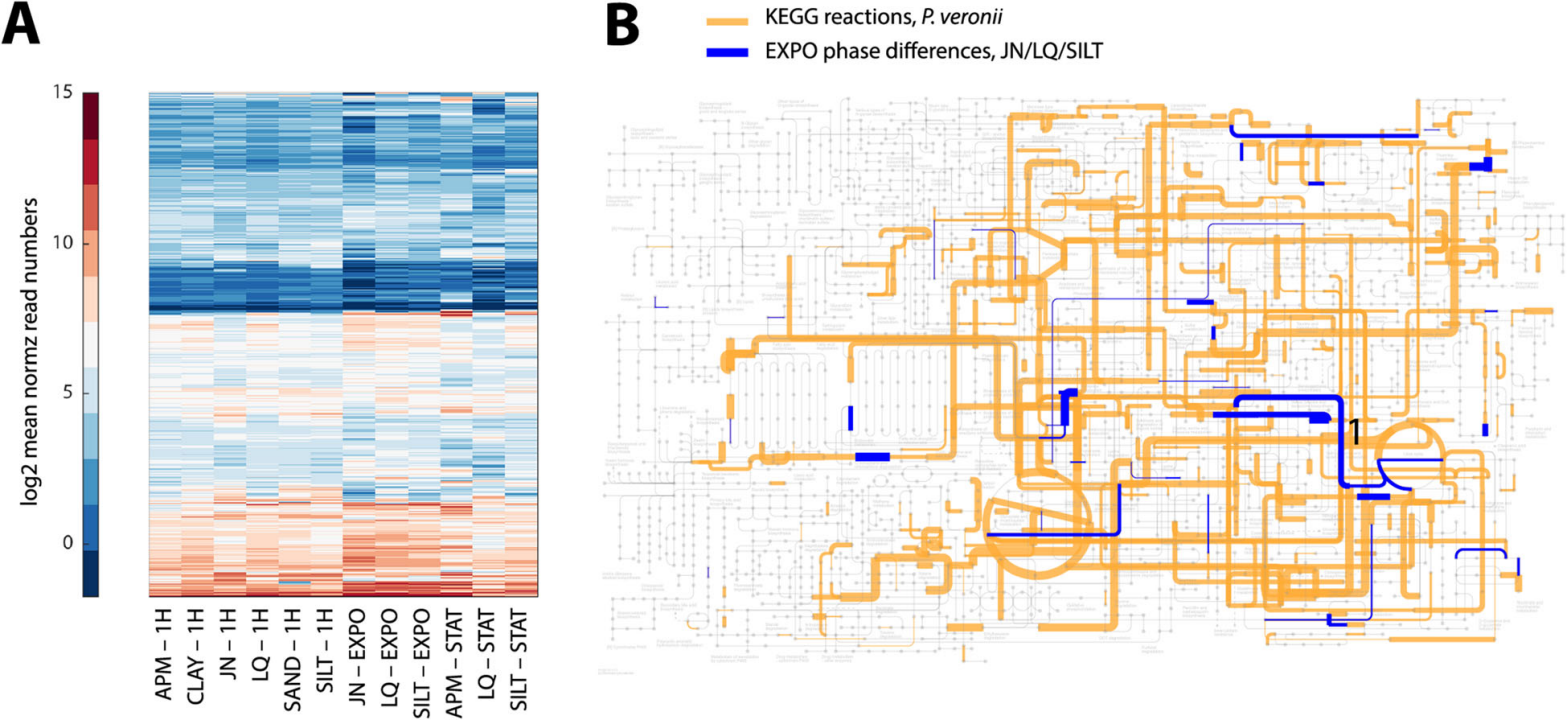
Expression of wild-type *P. veronii* core metabolic functions in different materials and conditions. **a** Log_2_-mean normalized read numbers of 1234 identified genes of the core metabolic network per 3–4 replicates and condition, clustered to expression levels and grouped according to growth phase (LAG, 1H; EXPO, STAT). **b** Mean expression levels of metabolic core functions in exponentially growing cells in Jonction (JN) plotted on the appropriate KEGG metabolic map using iPath3, with line width proportional to the log_2_ mean read numbers. In blue, those 72 reactions with significantly changed expression levels (i.e., > 2 × *SD* from the group mean) among JN, liquid (LQ) and Silt-grown cells (Supplementary Table 4)

Analysis of the inferred gene sets to be involved in metabolic reactions of *P. veronii* mappable to the KEGG database [22], revealed relatively coherent clustering among gene groups but more loose growth phase and sample clustering (Fig. 7a). This indicated relatively few changes within the core metabolism despite different growth environments. In particular, only 72 metabolic reactions (out of 1234 identified reactions using the iPsvr metabolic scale model [23]) showed outlier behaviour among the three exponential phase datasets (Fig. 7b, Supplementary Table 4). The rest of the metabolic network remained remarkably consistent and similar, given the different growth environments (liquid, Jonction, and Silt; Fig. 7b). These 72 reactions occur seemingly arbitrarily within the complete metabolic network, except for a notable pathway expression change in soils connected to the urea cycle (Fig. 7b, ‘1’). Remarkably, they are two-fold enriched for reactions implicating NADH/NAD^+^ compared to the total metabolic network (Supplementary Table 5), with mostly opposite expression changes between liquid or soil conditions (Jonction and Silt, Supplementary Table 4). This might indicate that cells in soils, although they grow at the same rates using essentially the same metabolic pathways as in liquid, replenish NADH/NAD^+^ by activating different side reactions, which they might need for e.g., defense mechanisms.

### Condition-specific and commonly expressed genes in stationary phase

Finally, we studied the global responses of *P. veronii* during stationary phase conditions and different growth environments. In comparison to 1 h, cells sampled in stationary phase conditions (Table 2) showed a small number of commonly differentially expressed genes without clear signature (Fig. 6c, Supplementary Table 6). In comparison to LQ, cells in stationary phase conditions in APM or Silt shared 248 higher and 79 lower expressed genes (Fig. 6c, Supplementary Data 26). Among the higher expressed genes were those of the *dmp* cluster and urea catabolism, indicating that cells in Silt or APM were still metabolizing aromatic substrates at that time of sampling (Supplementary Table 7). There was also evidence for increased synthesis of carbon and energy reserve polymers as known from other pseudomonads [45, 46], such as alginate (*alg*), glycogen (*glg*), and polyhydroxyalkanoates (*phaG*), the latter being induced up to 55-fold in APM and 200-fold in Silt (Supplementary Table 7).

Several transport systems were induced in Silt and APM in stationary phase (Supplementary Table 7), such as for amino acids (*ydh, bra*), putrescine (*spuD*), spermidine (*spuE*), nitrate (*nasA*), and sulfate (PVE_r1g3919). Genes associated with resistance mechanisms were higher expressed, for example, organic hydroperoxide resistance protein (*ohr*), and mercury resistance (*merACTP)*. Interestingly, several signaling genes such as *cheA*, *cheB2*, *cheW,* and *cheY*, PVE_r1g893, and PVE_r1g2555 were also higher expressed in APM and Silt compared to LQ (Supplementary Table 7). Homologs of those have been implicated in biofilm growth and flagella-mediated twitching [47]. These results indicated that *P. veronii* cells in stationary phase in porous environments remained metabolically active, diverting resources from growth towards synthesizing amino acids and reserve materials to promote survival and attachment.

## Discussion

The success of colonization of bacterial inoculants in soil depends on their metabolic and adaptive flexibility encoded in the genome, in broad relation to the environmental conditions of the soil itself, availability of substrates and nutrients, and its prevailent biological factors, such as background microbiota, phages or predators [15]. We show how adaptation, growth, and survival of *P. veronii* under soil conditions requires adjustment of a wide set of metabolic processes, ranging from nutrient requirements and carbon availability, motility and attachment, to respiration, trace metals, and defense against toxicity. By comparing transcriptomic responses in different soils and materials, and at different growth phases, we are confident to have covered a broad range of conditions that help our understanding of the mechanisms necessary for strain adaptation and survival upon inoculation.

Previous studies had indicated the massive difference in gene expression of *S. wittichii* RW1 growing in soil and liquid on dibenzofuran, suggesting that there may be something like a ‘soil’-specific transcriptome [48]. Furthermore, we previously studied global responses of three different bacteria taxa (*S. wittichii* RW1, *Arthrobacter chlorophenolicus* A1, and *P. veronii*) in controlled conditions of growth under sublethal solute and matric stress that mimicked water stress in soils, in order to identify common strategies [17]. By including inert silica particles (APM) here, we could see that a porous matrix by itself causes a global response that is to some extent similar to water stress imposed by the addition of solutes or inert swelling agents (matric stress) [17, 49, 50]. Notably, incubation in APM similarly triggered cellular osmoregulation, amino acid recycling and oxidative stress, together with reduced growth, which therefore seem to originate from perceived water stress. It is further worth mentioning that half of the genes that were specifically induced upon entry into porous medium encode hypothetical functions. Even though their function is unknown, they might correspond to a set of genes with important roles in general adaptation to and growth in porous media [17]. On the other hand, the *P. veronii* transcriptomic response observed here in APM and, previously, under imposed matric and solute stresses [17], was different from those in the soils. There is thus a response to soil different from that of porosity alone. Unexpectedly, growth of *P. veronii* with toluene in APM was worse than in liquid or natural soils, suggesting that porous conditions caused differences in the regulation of toluene metabolism in *P. veronii*.

One of the aims of this work was to study the effect of soil type on physiological responses and growth of *P. veronii*, and to understand if there are soil factors that would determine its adaptation and survival. The soils we worked with here have different microbiota background levels, and some showed growth on toluene, which may have invoked substrate competition on the introduced *P. veronii* cells. Unfortunately, we do not understand the nature of signs for competition in the *P. veronii* transcriptome. Soils further varied in pH, organic carbon content, structures and textures, leading to expected differences in e.g., substrate and water availability, matric stress, oxygen fluxes [51], or protozoan activity [38]. Indeed, upon soil inoculation, we observed the onset of nitrate respiration by *P. veronii* and induction of cytochrome c oxidases *cbb*_*3*_, which have been observed as low oxygen environmental responses of *P. aeruginosa* [52], *Pseudomonas stutzeri* A15, and *Pseudomonas fluorescens* YT01 [53, 54]. Nitrate respiration may confer a fitness advantage to *P. veronii* to adapt to soil microniches with low oxygen concentrations, as previous studies in controlled porous matrices have shown [55]. Possibly, nitrate respiration under low oxygen could be beneficial for toluene metabolism, to divert oxygen to the necessary dioxygenases for toluene breakdown and maintain respiratory energy generation from nitrate as electron acceptor. Adaptation in each soil and condition was accompanied by the selective induction of a variety of transporter systems (such as permeases, porins, multidrug transporters, amino acid ABC transporters). This illustrates the metabolic versatility of *P. veronii* to adapt to living in soil.

Regardless of the soil type and even in contaminated material such as Jonction, transition caused *P. veronii* cells to induce an osmoprotective response (putrescine and potassium uptake), to induce genes for exopolysaccharide production, and to regulate genes for copper homeostasis, indicating the importance of these processes for the initial soil adaptation. Of particular interest is the strong upregulation of systems involved in putrescine uptake and metabolism. The roles of putrescine (and other polyamines) are manifold, having been described both as signals for and during carbon or nitrogen starvation, regulation and oxidative stress defense [39], and recycling and metabolism of arginine [41]. The accumulation of compatible solutes such as putrescine (from which glutamate can be produced) and the uptake of potassium have been reported as the strongest “osmoprotectant strategies” employed by environmental bacteria to balance osmotic differences caused by the alteration in the solute potential of the extracellular environment [56]. Our data showed that inoculation into soil causes osmotic imbalance for *P. veronii*, whose reaction is in line with the three classical physiological responses to osmotic stress: polyamine transport and accumulation of glutamate and potassium [56]. Particularly in Clay, we observed the induction of alginate synthesis, which also has been proposed as an osmoadaptation mechanism of pseudomonads in environments with high osmolality [57, 58].

Importantly, we found that the *P. veronii* population developed in all non-sterile environments, more extensively when toluene was added as specific growth substrate, but also in its absence. This is in contrast to the much cited incapacity of inoculants to grow and be metabolically active in natural non-sterile materials, which may lead to bioaugmentation failures [9–11]. However, other reports have indicated adaptation, growth and survival of inoculants purposed for bioremediation at least in realistic non-sterile microcosms [48, 59–61]. We acknowledge that microcosms are not the same as field experiments, but at least this shows that many bacteria with potentially interesting properties for bioremediation are capable of establishing in complex microbial ecosystems. Those studies and including our own results indicated strain level adaptation to the specific environment (of the microcosm) and the carbon source [48, 59–61]. The variety of different responses, unfortunately, precludes some sort of global ‘one-for-all’ interpretation of adaptive characters that inoculants would need to have in order to be ‘fit’ for their intended deployment, except a recurrent signature of differentially expressed chemotactic and flagellar biosynthesis genes. We aimed to find additional conditions under which *P. veronii* would not be able to adapt and proliferate, but unfortunately, we could not really exploit this at transcriptomic level. Growth in Jonction material without added toluene was clearly not favorable for *P. veronii*, but the transcriptomic response from 1 h exposure in Jonction with toluene (in which it could grow) did not particularly show signs of poor adaptation. Except for increased induction of stress defense systems and changes in expression of genes for respiratory activity, there were no particular signs of physiological breakdown in cells in Jonction. The only other environment, which did not lead to strong population development of *P. veronii* on toluene was Clay. We think that the most likely causes for population loss here were predation and, possibly, competition of native soil microbiota for toluene. Unfortunately, we did not manage to isolate sufficient RNA for transcriptome analysis from *P. veronii* during exponential growth in Clay. However, during the 1-h transition in Clay we observed a decrease of expression of genes for flagellar assembly and motility, which was not present in Silt or Sand. Flagella allow bacteria to explore their environment, to search for nutrients and to escape from predators or adverse conditions, but activation of flagellar genes have also been implicated in the solvent stress tolerance response, where the flagellar export apparatus is used to export other proteins unrelated to flagellar assembly [62]. Overall, the role of flagella synthesis during soil adaptation remains unclear. Growing *S. wittichii* RW1 in sand [48], or clay with dibenzofuran [60] diminished expression of flagella synthesis genes. Both *S. wittichii* RW1, *Artrobacter chlorophenolicus* A6 and *P. veronii* reduce expression of flagella synthesis under solute and matric stress [17], and similar behaviour has been detected in other bacteria under water stress [58, 63]. In contrast, *P. putida* KT2440 exposed to water stress on ceramic plates did not show significant difference in flagellar gene expression [49], whereas *P. veronii* during growth in Silt with toluene upregulated flagellar gene synthesis (Table 5). This contrasting behaviour may indicate strain-specific finetuning of flagellar expression in relation to available energy levels. Perhaps, therefore, the diminished expression of *P. veronii* flagellar genes during the 1-h adaptation to Clay was a result of redirected energy resources. The resulting reduced cell motility could then have favored grazing by protozoa.

The analysis of gene expression during growth of *P. veronii* allowed us to identify further differences in soils versus liquid. It has been reported that in bulk soils carbon rather than nutrients such as N, S, P, and Fe is the limiting factor for bacteria growth and fitness [64, 65]. In contrast, in hydrocarbon- or PAH-polluted soils the addition of macro- (N, P, S, K) and micro-elements (Fe) is usually practiced to enhance bacterial activity, because of the excess carbon posed by the pollution [66–69]. Expression of functions related to nutrient scavenging by *P. veronii* cells in soils suggests that they quickly perceive nutrient limitation and can adapt to some extent. For example, the observed higher expression of genes involved in sarcosine recycling may have been a response to limiting nitrogen. In addition, perceived nitrogen limitation may have led to increased turnover of branched-chain amino acids and asparagine, plus leading to different usage of the urea pathways. Growth in soils may also have led to sulfur limitation and to induction of increased assimilation of sulfite from sulfate, and catabolism of cysteine and taurine. These examples indicate that availability of nutrients is important for establishment of inoculants in polluted soils.

## Conclusion

In conclusion, our study clearly showed *P. veronii* adaptation, growth, and survival in different non-sterile soils and contaminated material. Although we externally added toluene as a specific carbon and energy source for the proliferation of *P. veronii*, its previously observed abundance and activity in contaminated sites [19, 70] indicate its capacity to survive under field conditions. These are important observations because they contradict the regular notion of poor growth and survival for exogenous strains in complex microbial ecosystems [71], and thus, provide basis to better select inoculants for applications in bioaugmentation. Under the tested conditions, we did not find a single “soil-transcriptomic” program but instead identified and highlighted both a core of commonly as well as specifically induced functions (many of which consist of uncharacterized proteins) in soils that contribute to the strain’s adaptation under different conditions. The strain expressed a remarkably robust metabolic program during growth, which was maintained irrespectively of its environment. We did not identify critical factors associated with the failure of the strain upon inoculation beyond potential predation, possible substrate competition and signs of nutrient limitations in later growth phases.

At this point, our comprehension (and that of many others) is necessarily a ‘narrative’ of understanding how cells adapt and grow, concluded from the conglomerate of global (e.g., GO and COG) analysis as well as that of individual gene annotations. However, specific cellular reactions are clearly different between typical liquid cultures and in soil. Therefore, it seems crucial to us to study strain behaviour under the conditions of the expected complex environments (i.e., soil, gut, skin), and not in standard liquid culture. In the future, this knowledge may help to better predict the success of inoculants.

## Supplementary Information

**Additional file 1: Supplementary Figure 1.** Comparison of growth rates on toluene of *P. veronii* wild-type and the *P. veronii* gfp-tagged variant. **Supplementary Figure 2.** Background soil microbiota counts. **Supplementary Figure 3.** Cell washing recoveries of wild-type *P. veronii* cells inoculated to soils. **Supplementary Figure 4.** Background growth of *P. veronii* miniTn*5::gfp* on soil organic carbon or soil microbiota on toluene.

**Additional file 2: Table S1.** Summary of total mapped reads of inoculated microcosms exposed to toluene. **Table S2.** Subset of genes whose expression levels responded to inoculation into glass beads (APM) versus liquid (FDR < 0.05). **Table S3.** Subset differentially expressed genes of *P. veronii* 1YdBTEX2 in all soils vs controls (Liquid&APM). **Table S4.** Relevant KEGG reactions with outlier expression in exponential phase conditions. **Table S5.** Biochemical term enrichment in the exponential phase outlier reactions compared to all metabolic reactions. **Table S6.** Commonly differentially expressed genes in Liquid, glass beads (APM) and Silt in stationary phase. **Table S7.** Subset of commonly differentially expressed genes in glass beads (APM) and Silt in stationary phase.

**Additional file 3: DATA 1.** Complete list of differentially expressed genes in APM-1H versus liquid-1H (FDR < 0.05). **DATA 2.** Enriched GO terms among the significantly differentially downregulated genes in cells growing on glass beads (APM) versus liquid media after 1 h inoculation. **DATA 3.** Complete list of differentially expressed genes in Sand-1H versus APM-1H (FDR < 0.05). **DATA 4.** Complete list of differentially expressed genes in Silt -1H versus APM-1H (FDR < 0.05). **DATA 5.** Complete list of differentially expressed genes in Clay-1H versus APM-1H (FDR < 0.05). **DATA 6.** Enriched GO terms among the significantly differentially upregulated genes in Sand versus APM after 1 h contact. **DATA 7.** Enriched GO terms among the significantly differentially expressed upregulated genes in Silt versus glass beads (APM) after 1 h contact. **DATA 8.** Enriched GO terms among the significantly differentially expressed downregulated genes in Silt versus glass beads (APM) after 1 h contact. **DATA 9.** Enriched GO terms among the significantly differentially expressed upregulated genes in Clay versus glass beads (APM) after 1 h contact. **DATA 9.** Enriched GO terms among the significantly differentially expressed downregulated genes in Clay versus glass beads (APM) after 1 h contact. **DATA 11.** Differentially expressed genes in all natural soils-1H vs controls-1 h (ANOVA. FDR < 0.05). **DATA 12.** Complete list of differentially expressed genes in Jonction-1H vs APM-1H (FDR < 0.05). **DATA 13.** Commonly differentially expressed genes in soils and Jonction soils after 1 h contact versus control conditions -1H (ANOVA. FDR < 0.05). **DATA 14.** Unique differentially expressed genes in Jonction-1H vs APM-1H. **DATA 15.** Enriched GO terms among the significantly differentially expressed upregulated genes in Jonction versus glass beads (APM) after 1 h contact. **DATA 16.** Enriched GO terms among the significantly differentially expressed downregulated genes in Jonction versus glass beads (APM) after 1 h contact. **DATA 17.** Comonly differentially expressed genes in *Pseudomonas veronii *cells growing exponentialy in Liquid, Sand and Silt vs same material -1H (FDR < 0.05). **DATA 18.** Enriched GO terms among the significantly differentially expressed upregulated genes in LIQUID-EXPO vs LIQUID-1 h. **DATA 19**. Enriched GO terms among the significantly differentially expressed downregulated genes in LIQUID-EXPO vs LIQUID-1 h. **DATA 20.** Enriched GO terms among the significantly differentially expressed upregulated genes in SILT-EXPO vs SILT-1 h. **DATA 21.** Enriched GO terms among the significantly differentially expressed downregulated genes in SILT-EXPO vs SILT-1 h. **DATA 22.** Enriched GO terms among the significantly differentially expressed upregulated genes in JONCTION-EXPO vs JONCTION-1 h. **DATA 23.** Enriched GO terms among the significantly differentially expressed downregulated genes in JONCTION-EXPO vs JONCTION-1 h. **DATA 24.** Complete list of differentially expressed genes in SILT-EXPO vs SILT-1H (FDR < 0.05). **DATA 25.** Complete list of differentially expressed genes in Jonction-EXPO vs JN-1H (FDR < 0.05). **DATA 26.** Commonly differentially express genes in APM and Silt in stationary phase (FDR < 0.05). **DATA27.** Unique differentially express genes Silt in stationary phase (FDR < 0.05). **DATA 28.** Unique differentially express genes in APM-STAT vs APM-1H (FDR < 0.05). **DATA 29.** Enriched GO terms among the significantly differentially expressed upregulated genes in SILT-STAT vs SILT-1 h. **DATA 30.** Enriched GO terms among the significantly differentially expressed downregulated genes in SILT-STAT vs SILT-1 h. **DATA 31.** Enriched GO terms among the significantly differentially expressed upregulated genes in AS-STAT vs AS-1 h . **DATA 32.** Enriched GO terms among the significantly differentially expressed downregulated genes in AS-STAT vs AS-1 h. **DATA.** Enriched GO terms among the significantly differentially expressed upegulated genes in LIQUID-STAT vs LIQUID-1 h. **DATA.** Enriched GO terms among the significantly differentially expressed downregulated genes in LIQUID-STAT vs LIQUID-1 h.

## Data Availability

The raw sequence reads of the RNA-seq data sets are available from the Short Read Archive under Bioproject number PRJNA682712.

## Abbreviations

EXPO: Exponential growth phase
STAT: Stationary phase
RNA-seq: Reverse-transcribed ribosomal RNA depleted library sequencing
GO: Gene ontology
COG: Cluster of orthologous groups
CFUs: Colony-forming units
GWC: Gravimetric water content
APM: Artificial porous matrix
OD: Optical density (culture turbidity)
FDR: False discovery rate
