# Supplementary material for: Genome-wide gene expression changes of Pseudomonas veronii 1YdBTEX2 during bioaugmentation in polluted soils
Source: Environ Microbiome. 2021 Apr 29;16:8. doi: 10.1186/s40793-021-00378-x (PMC8082905; doi:10.1186/s40793-021-00378-x)
Supplement: Supplementary file 1 — Additional file 1: Supplementary Figure 1. Comparison of growth rates on toluene of P. veronii wild-type and the P. veronii gfp-tagged variant. Supplementary Figure 2. Background soil microbiota counts. Supplementary Figure 3. Cell washing recoveries of wild-type P. veronii cells inoculated to soils. Supplementary Figure 4. Background growth of P. veronii miniTn5::gfp on soil organic carbon or soil microbiota on toluene. [file 40793_2021_378_MOESM1_ESM.pdf]

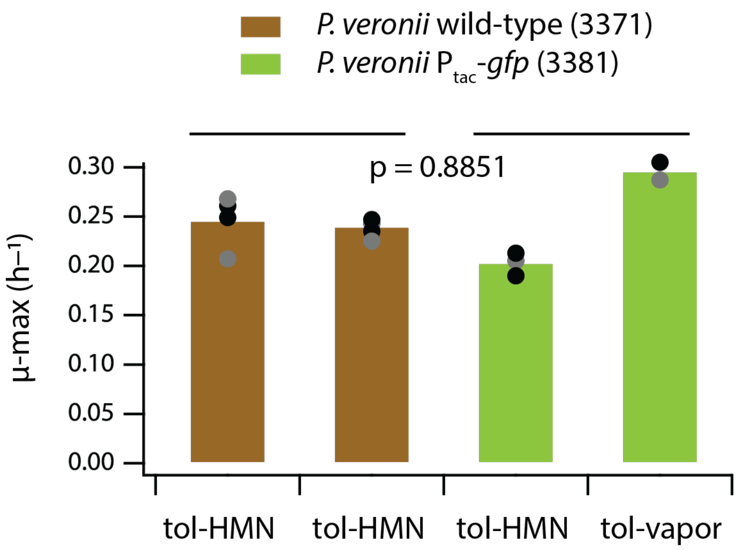

Supplementary figure 1. Comparison of growth rates on toluene of *P. veronii* wild-type and the *P. veronii* gfp-tagged variant.

Bars show the mean maximum growth rate derived from the slope of the $\log_{10}$ population counts (CFU per ml) versus time (h). Data points show values from the individual replicates (*n = 2 – 4*). In green, gfp-tagged variant (strain 3381); in brown, wild-type variant (strain 3371). Tol-HMN, toluene addition through a secondary phase of heptamethylnonane; tol-vapor, toluene addition through the vapor phase. P, adjusted p from strain-level comparison groups in Bartlett test, followed by post-Hukey test.

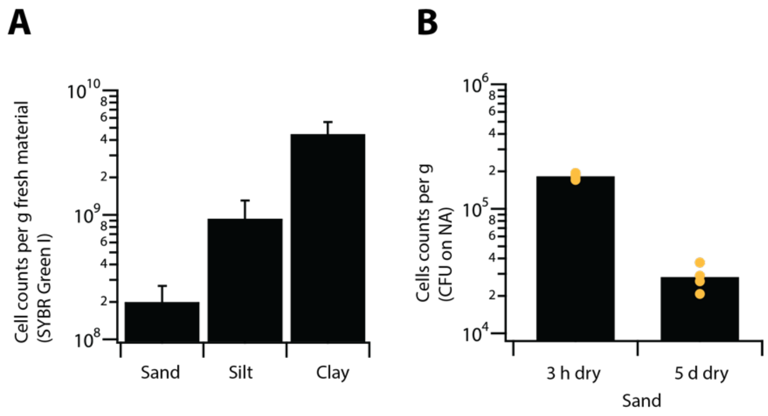

Supplementary figure 2. Background soil microbiota counts.

A) Mean cell counts per gram fresh material ± one *SD* (as SYBR Green I fluorescent cells) of Sand, Silt and Clay.

B) Mean cell counts per g material (as colony forming units on nutrient agar) of Sand after 3 h and 5 d ambient air temperature drying. Dots show individual replicate values (*n = 4*).

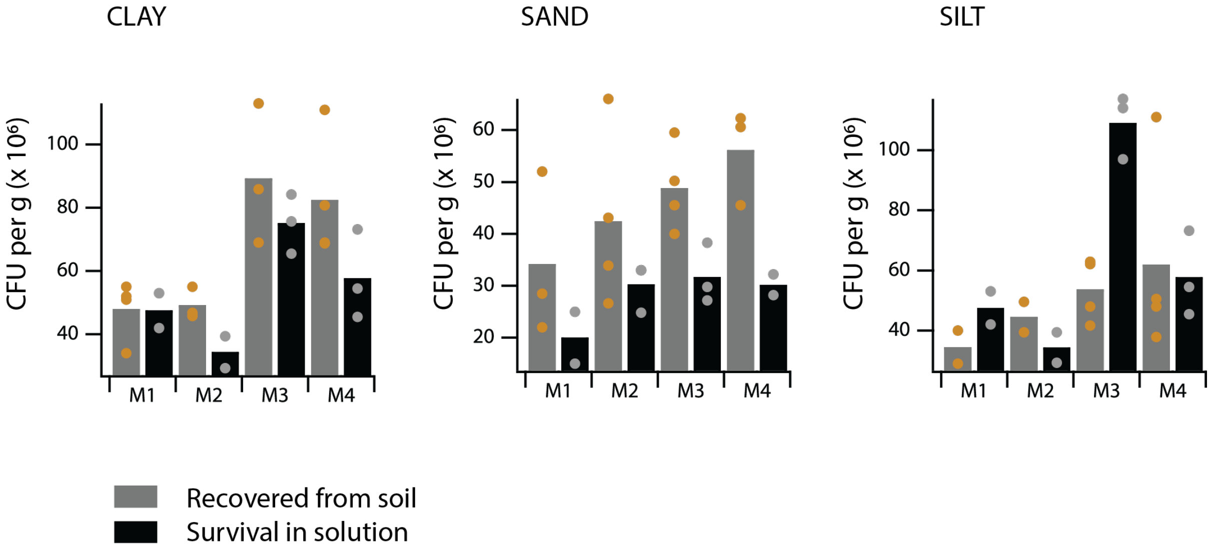

Supplementary figure 3. Cell washing recoveries of wild-type *P. veronii* cells inoculated to soils.
Diagrams show population sizes (Bars = mean, dots = individual replicates) in colony forming units (CFU) per g recovered from soil 1 h after addition (grey bars) in comparison to the same *P. veronii* population left in liquid suspension (black bars). Three soil types were tested, each with different added cell densities.

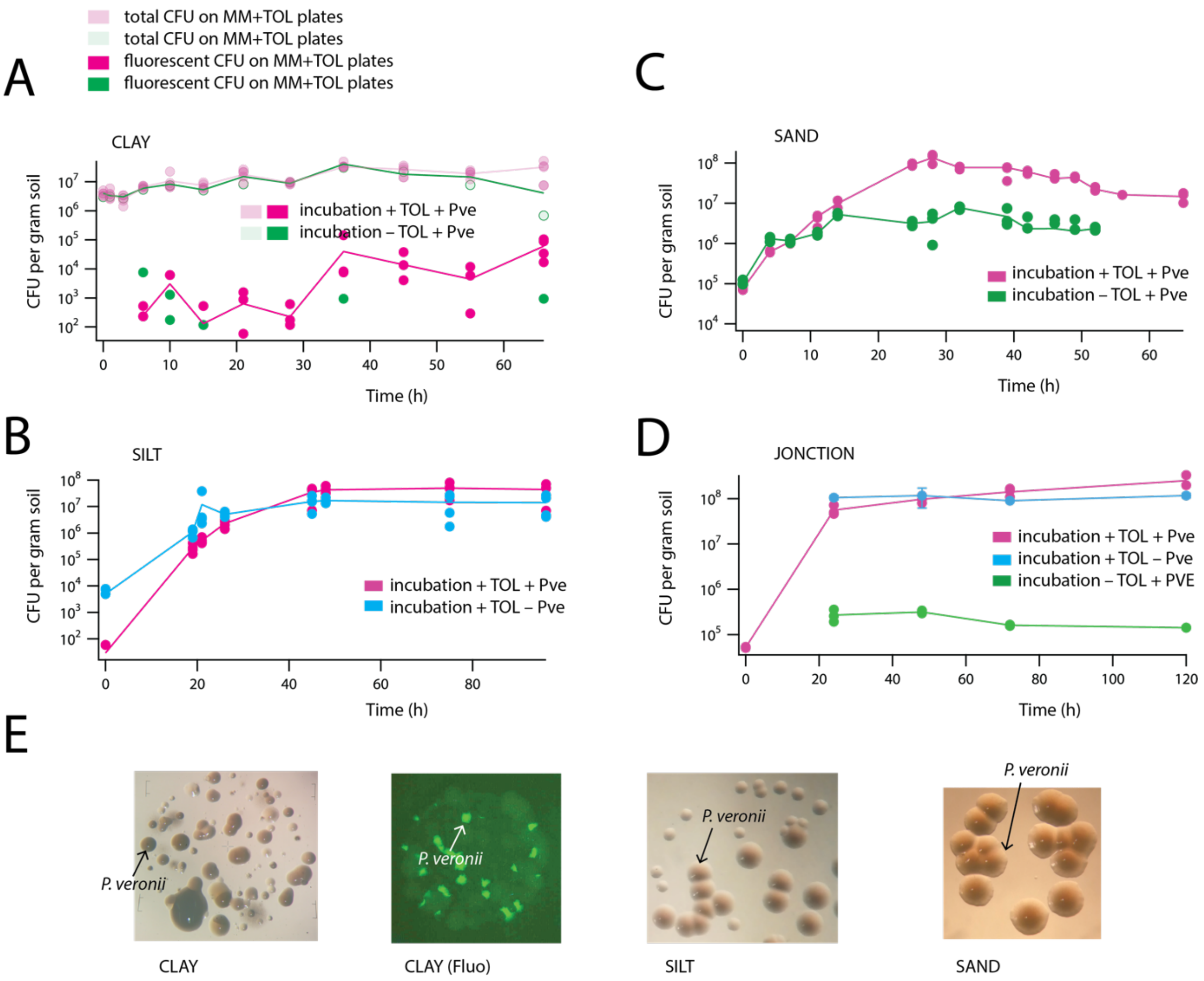

Supplementary figure 4. Background growth of *P. veronii* miniTn*5::gfp* on soil organic carbon or soil microbiota on toluene.

A) Community sizes (in colony forming units, CFU per gram soil), deduced from growth on agar plates with toluene supplied through the gas phase (MM+TOL plates). Clay microcosms inoculated with *P. veronii* (Pve) and incubated in presence (+TOL, magenta) or absence (–TOL, green) of toluene vapor. Transparent symbols, total counts of colonies on MM+TOL plates. Solid symbols, counts of colonies that were additionally verified for GFP fluorescence (hence, gfp-tagged Pve).

B) as A but for silt. Blue symbols, microcosms without Pve inoculation but incubated with toluene vapor.

C) as A but for sand. No background growth of soil microbiota on MM+TOL plates was detected.

D) as A but for Jonction (sieved mixture as defined in the main text).

E) Examples of appearing colonies on MM plates incubated with toluene vapor. Clay (Fluo), fluorescent colonies of *P. veronii* amidst others (not the same plate area as in Clay).

Lines are the means and filled symbols are individual biological replicate values.
